# Genome-wide pQTL analysis of protein expression regulatory networks in the human liver

**DOI:** 10.1186/s12915-020-00830-3

**Published:** 2020-08-10

**Authors:** Bing He, Jian Shi, Xinwen Wang, Hui Jiang, Hao-Jie Zhu

**Affiliations:** 1 Department of Clinical Pharmacy, University of Michigan College of Pharmacy, 428 Church Street, Room 4565 NUB, Ann Arbor, MI 48109-1065 USA; 2 Department of Biostatistics, University of Michigan, Ann Arbor, MI 48109 USA

**Keywords:** Proteomics, Protein expression regulation, Protein quantitative trait loci, Protein-disease interaction

## Abstract

**Background:**

Previous expression quantitative trait loci (eQTL) studies have identified thousands of genetic variants to be associated with gene expression at the mRNA level in the human liver. However, protein expression often correlates poorly with mRNA levels. Thus, protein quantitative trait loci (pQTL) study is required to identify genetic variants that regulate protein expression in human livers.

**Results:**

We conducted a genome-wide pQTL study in 287 normal human liver samples and identified 900 local pQTL variants and 4026 distant pQTL variants. We further discovered 53 genome hotspots of pQTL variants. Transcriptional region mapping analysis showed that 1133 pQTL variants are in transcriptional regulatory regions. Genomic region enrichment analysis of the identified pQTL variants revealed 804 potential regulatory interactions among 595 predicted regulators (e.g., non-coding RNAs) and 394 proteins. Moreover, pQTL variants and trait-variant integration analysis implied several novel mechanisms underlying the relationships between protein expression and liver diseases, such as alcohol dependence. Notably, over 2000 of the identified pQTL variants have not been reported in previous eQTL studies, suggesting extensive involvement of genetic polymorphisms in post-transcriptional regulation of protein expression in human livers.

**Conclusions:**

We have partially established protein expression regulation networks in human livers and generated a wealth of pQTL data that could serve as a valuable resource for the scientific community.

## Introduction

High-throughput sequencing technologies enabled the analysis of genome-wide expression quantitative trait loci (eQTL) to study the transcriptional and post-transcriptional regulatory mechanisms involved in the regulation of mRNA expression [1]. Tens of thousands of eQTL variants have been identified to be associated with mRNA expression in the human liver [2]. However, mRNA expression correlates poorly with protein levels for many genes [3], which is in part due to many post-transcriptional factors, such as sequence features implicated in protein translation and degradation [4, 5]. Chick et al. studied genome-wide mRNA and protein expression in 192 mouse livers and discovered that only half of the identified protein quantitative trait loci (pQTL) were also eQTLs [6]. Such a discrepancy between eQTLs and pQTLs was also observed in a proteomics study of liver mitochondria in recombinant inbred mice [7]. Several eQTL studies have been performed in human livers [8–11], but the discrepancy between mRNA and protein expression necessitates a further study of hepatic gene expression regulation at the protein level. However, genome-wide pQTL studies and associated network analyses have not been conducted in human livers.

**Fig. 1.**
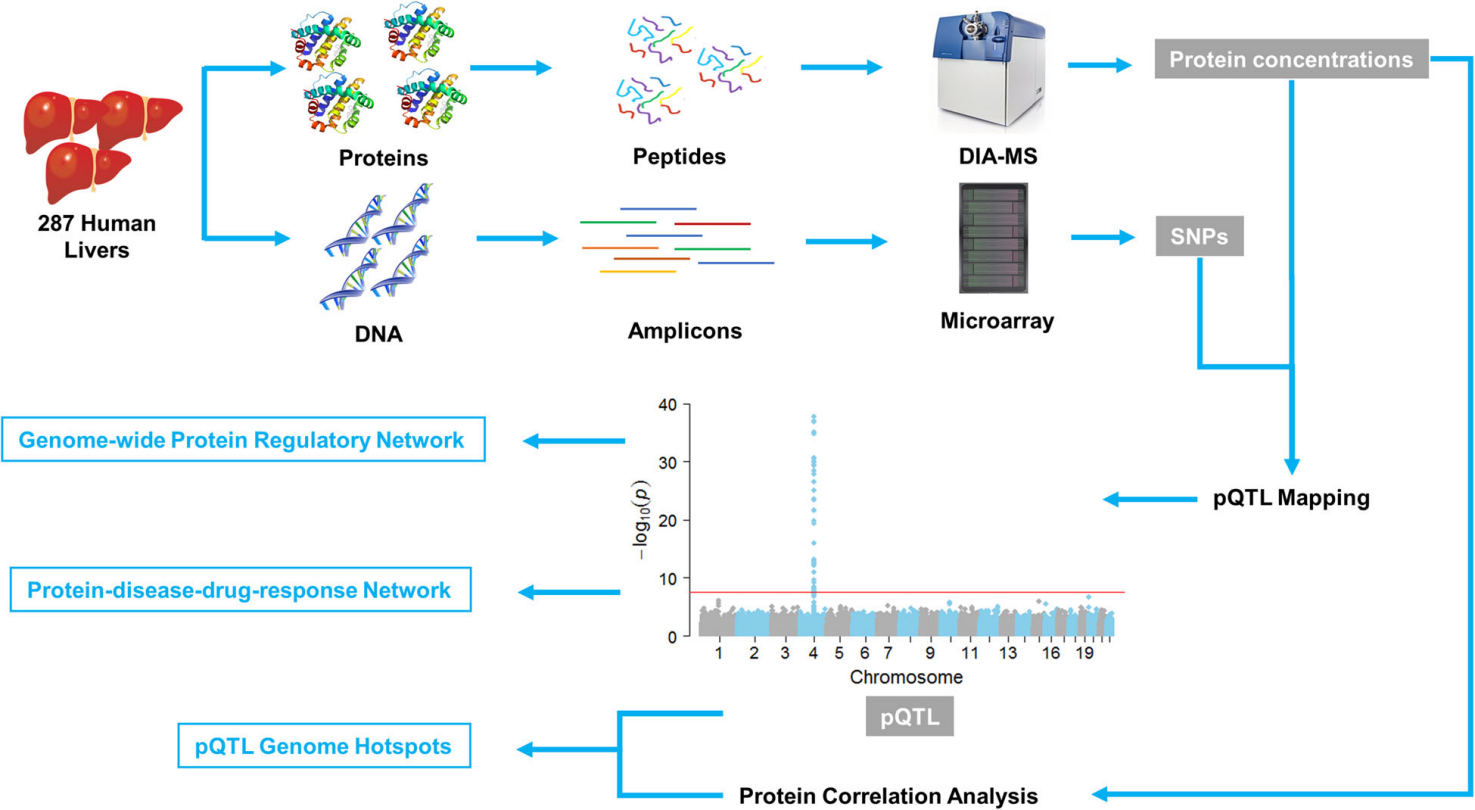
A schematic diagram of the study investigating protein expression regulation networks in human livers. We performed genome genotyping and whole proteome absolute protein quantification in 287 human liver samples. Transcriptional regulatory region mapping and genomic region enrichment analysis were conducted to uncover protein expression regulatory networks. We also identified protein-disease-drug response networks and pQTL genome hotspots

Liquid chromatography-tandem mass spectrometry (LC-MS/MS)-based proteomics is a powerful approach for relative and absolute quantification of targeted proteins and of proteins at the global scale [12]. Compared to relative quantification, absolute protein quantification (APQ) is often more desirable for revealing complex protein expression regulation networks [13]. Label-free APQ methods are not dependent on heavy isotope-labeled internal standard proteins or peptides and can be used to quantify a large number of proteins [14]. However, most label-free APQ methods are based upon MS1 precursor ion signals obtained from data-dependent acquisition (DDA), which is biased towards highly abundant peptides [15]. Schubert and colleagues established a data-independent acquisition (DIA)-based APQ method using a linear correlation model built on a set of pre-selected anchor proteins [16]. We recently developed a label-free APQ method named DIA-TPA that uses MS2 intensity signals from DIA data and an improved total protein approach (TPA); this method enabled high-throughput global absolute protein quantification and was successfully used to absolutely quantify human liver proteomes [17]. In the present study, we conducted a global proteomic analysis in 287 normal human liver samples using DIA-TPA and analyzed protein-protein association patterns. Furthermore, we performed a genome-wide pQTL study to uncover both transcriptional and post-transcriptional mechanisms regulating protein expression in human livers (Fig. 1). The study also determined genome hotspots of pQTLs for correlated proteins.

## Results

### Absolute quantification and subcellular location of hepatic proteins

**Fig. 2.**
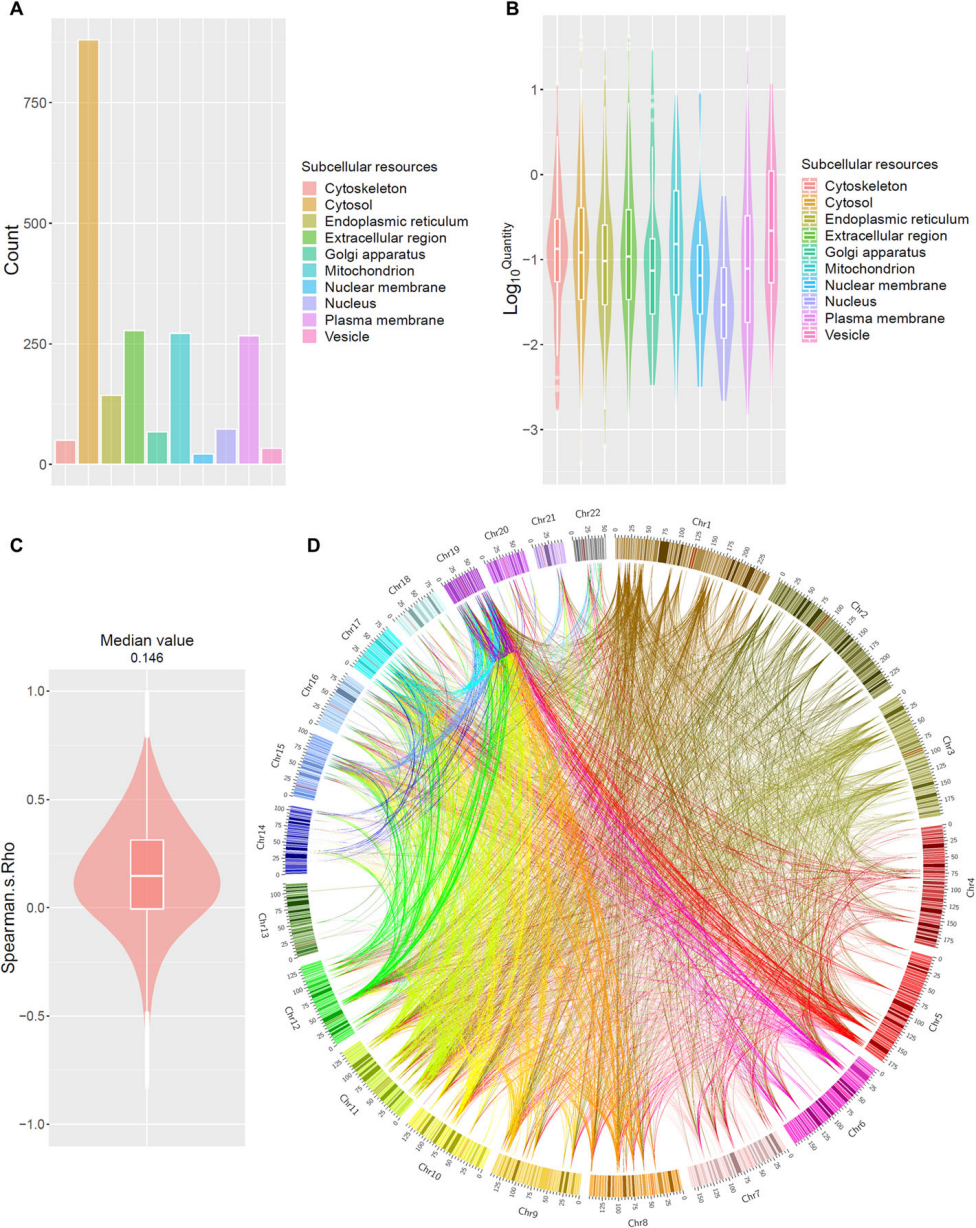
Features of quantified proteins in human livers. The number of quantified proteins in major subcellular locations (**a**). Violin plots of log10-transformed protein concentrations in major subcellular locations (**b**). The analysis was performed using protein subcellular location data obtained from the Gene Ontology (GO) database. Violin plot of Spearman’s correlations (Spearman’s rho) of protein expression levels in human liver (**c**). The medium value of Spearman’s rho was about 0.146. Circos plot of chromosome locations of highly correlated proteins (Spearman’s rho > 0.8 or < − 0.8) (**d**). Lines in the circos plot represent the correlations (Spearman’s rho > 0.8 or < − 0.8) between proteins. Colors of the lines represent chromosomal source of the correlated proteins. If proteins were from different chromosomes, the colors were determined by the chromosome with a smaller ID number

A total of 1508 proteins were absolutely quantified in 287 HLS9 samples using the DIA-TPA proteomic method (Additional file 1: Fig. S1 and Additional file 2: Data file S1). The number of proteins quantified in the present study is much less than the number of mRNA-expressing genes in human livers as documented in GTEx (1508 vs 26,560) [10], which is likely due to that many transcripts are not translated into proteins, and the concentrations of many proteins are below the limit of detection of our proteomics assay. The relatively small number of quantified proteins is also partially attributed to a less sensitive micro-flow LC setting adopted in this study and the use of a spectral library generated from the DDA analysis of pooled HLS9 samples without peptide fractionation. Subcellular location analysis indicated that these proteins originated from all major cellular components, ranging from the nucleus to the extracellular region (Fig. 2a). Cytosol was the largest source of proteins, containing about half of the quantified proteins. Proteins from different subcellular sources showed varied expression levels. For example, vesicle proteins exhibited the highest median abundance values, while nuclear proteins were the lowest (Fig. 2b). Human liver transcriptome data retrieved from the GTEx database revealed a different mRNA expression pattern (Additional file 1: Fig. S1A). Most proteins had similar expression patterns across samples, although interindividual variability existed (Additional file 3: Fig. S2). Therefore, we performed a correlation analysis of protein expression in the 287 human liver samples.

### Correlation of hepatic protein expression across individuals

Spearman’s correlation analysis indicated that there were more positively correlated proteins than negatively correlated. The median value of Spearman’s rho (correlation coefficient) of significantly correlated protein-protein pairs was 0.146 (Fig. 2c), which is lower than that (Spearman’s rho = 0.354) of the same genes at the transcript level in human livers, as reported in GTEx (Additional file 1: Fig. S1B). There were 104,357 medium-high correlations (i.e., Spearman’s rho > 0.5 or < − 0.5) involving 1358 proteins. Moreover, there were 4538 high correlations (Spearman’s rho > 0.8 or < − 0.8) encompassing 501 quantified proteins. Interestingly, the majority of the genes encoding for the highly correlated proteins (Spearman’s rho > 0.8 or < − 0.8) were located on different chromosomes (Fig. 2d), indicating that trans-regulation mechanisms may play a primary role in the regulation of protein co-expression in the liver.

### Genome-wide pQTL analysis

**Fig. 3.**
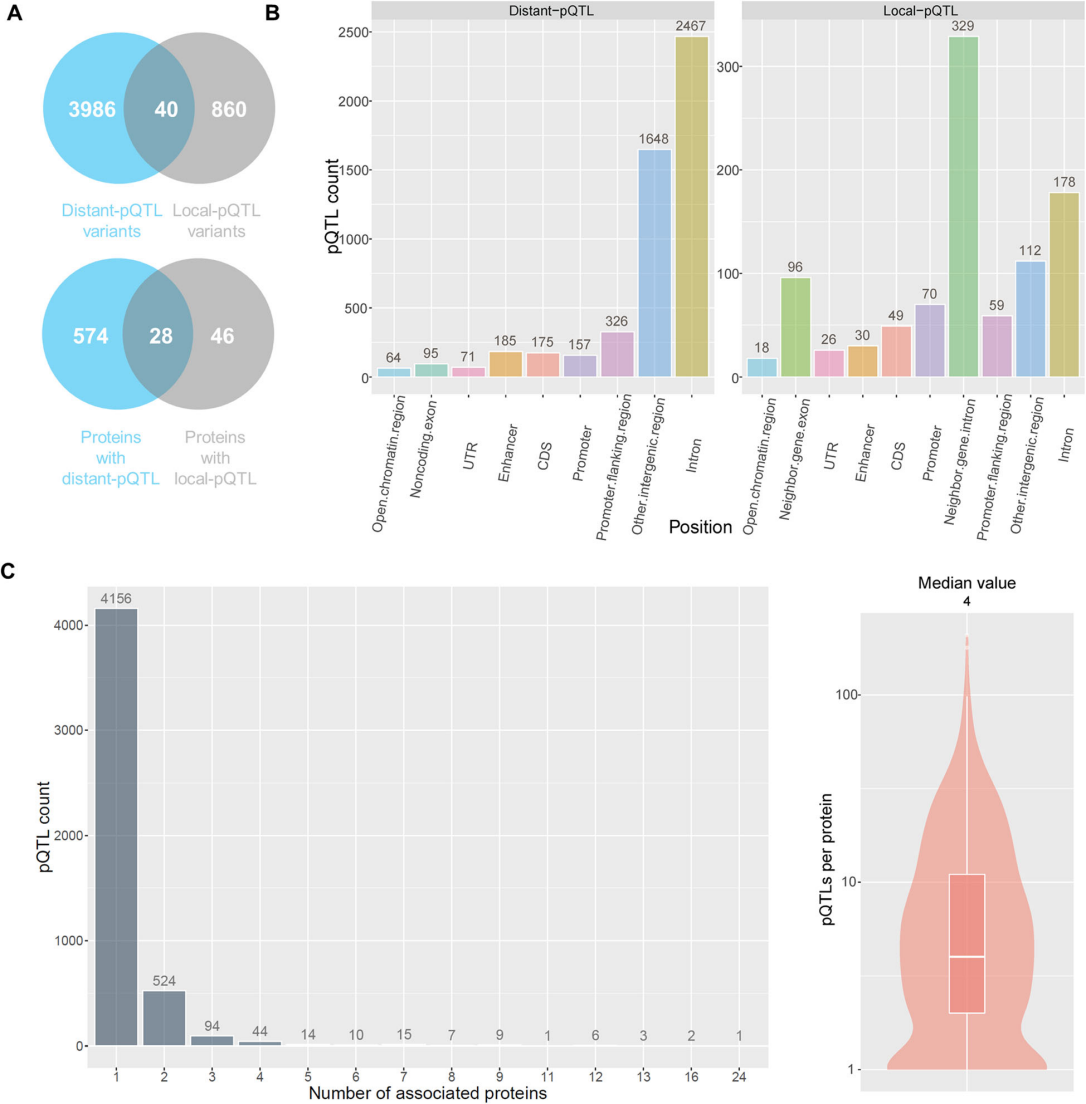
Statistics of pQTLs discovered in human livers. Venn plots of the identified local and distant pQTL variants (**a**, top) and proteins with local pQTLs and/or distant pQTLs (**a**, bottom). A pQTL variant associated with multiple proteins was counted once. Distribution of local and distant pQTL variants in genomic regions (**b**). The number placed on the top of a column indicates the number of pQTL variants found in the specific genomic region. A pQTL variant associated with the expression of multiple proteins can be counted multiple times. For example, if a local pQTL variant was associated with the expression of two proteins, and the variant was located in the CDS region of one protein and the promoter region of another protein, then the variant was counted twice as a local pQTL in both CDS and promoter regions. The number of pQTLs found to be associated with various number of proteins (**c**, left). Violin plot of the number of pQTL variants per protein (**c**, right). The median number of pQTL variants per protein was 4

To investigate the regulatory mechanisms of protein expression, we genotyped 287 human liver samples and performed a genome-wide pQTL analysis for the 1344 proteins identified in more than 90% of samples. We identified 6155 pQTL variant-protein interactions having a genome-wide significance (*p* value < 2.99 × 10^−8^), involving 4886 pQTL variants and 648 proteins (Additional file 4: Data file S2). The pQTL variants contained 2161 independent locus markers after LD pruning. Among the identified variants, 860 were local pQTL variants and 3986 were distant pQTL variants (Fig. 3a). In addition, 40 variants acted as either local or distant pQTLs for different proteins. Among the independent locus markers, 246 were local pQTLs, 1905 were distant pQTLs, and 10 acted as either local or distant pQTLs for different proteins. Among the proteins with significant pQTL variants, 46 and 574 proteins had only local pQTLs and distant pQTLs, respectively, while 28 proteins were associated with both local and distant pQTL variants (Fig. 3a). Beyond transcription regulatory regions, such as promoters and enhancers, pQTL variants were also found in post-transcription regulatory regions such as untranslated regions (UTRs) and coding sequence (CDS) (Fig. 3b) [4]. Surprisingly, local pQTL variants were mostly enriched within the intronic regions of the neighboring genes, while distant pQTL variants predominated in the introns of distant genes (Fig. 3b), which implies an important role for introns in the regulation of protein expression. Most pQTL variants were associated with the expression of a single protein, but 730 pQTL variants were associated with multiple proteins, including 67 local pQTL and 623 distant pQTL variants and 40 SNPs acting as both local and distant pQTLs (Fig. 3c, left; Additional file 5: Fig. S3). In an extreme case, the SNP rs78209928 was a pQTL variant for 24 proteins. Most proteins had more than one pQTL variant; the median number of pQTL variants per protein was 4 (Fig. 3c, right) (4 and 4.5 for distant and local pQTL variants, respectively) (Additional file 5: Fig. S3). The Spearman’s correlation between the effect size (beta value) and minor allele frequency (MAF) of pQTL variants was − 0.111, indicating that pQTL variants with lower MAF tend to have a greater impact on protein expression. The correlations between the beta value and MAF for coding, non-coding, local, and distant pQTL variants were − 0.163, − 0.109, − 0.246, and − 0.081, respectively.

We retrieved human liver eQTL results from four published datasets [8–11], including 12,481 eQTLs for 12,481 genes from Innocenti et al. [11], 1027 eQTL for 337 genes from Schroeder et al. [9], 6754 eQTL for 6089 genes from Schadt et al. [8], and 323,428 eQTL for 4000 genes from GTEx (version 7) [10]. The datasets contain both local eQTLs and distant eQTLs. Given that many eQTL SNPs were not identified in our study because of different genotyping arrays and populations, and protein levels of many previously reported regulated genes were under the detection limit of our proteomics assay, we filtered eQTLs by focusing on SNPs genotyped in both eQTL and pQTL studies and genes with both mRNA and protein quantifications. A total of 256 genes were found to have both eQTLs and pQTLs, while 266 and 392 genes had only eQTLs and pQTLs, respectively (Additional file 6: Fig. S4A). Across all genes, 1373 eQTL and 2750 pQTL associations were identified, but only 296 were shared associations found in both previous eQTL studies and the present pQTL investigation (Additional file 6: Fig. S4B). The shared associations contained 256 variants (Additional file 6: Fig. S4C), and all variants were local QTLs, which is consistent with a previous study and suggests that local QTLs tend to affect both mRNA and protein abundance [6]. Of note, most of the eQTLs were local eQTLs whereas the identified pQTLs were more evenly distributed between local and distant pQTL regions (Additional file 6: Fig. S4D-E).

We further analyzed the colocalization of primary pQTL and eQTL signals and found that the lead pQTL variants of GMPPB, QPRT, DECR2, ETHE1, HAAO, DDAH2, GSTA2, and CNDP2 were colocalized with the previously reported eQTL variants (Additional file 7: Table S1) [8–11].

### pQTL hotspot analysis

**Fig. 4.**
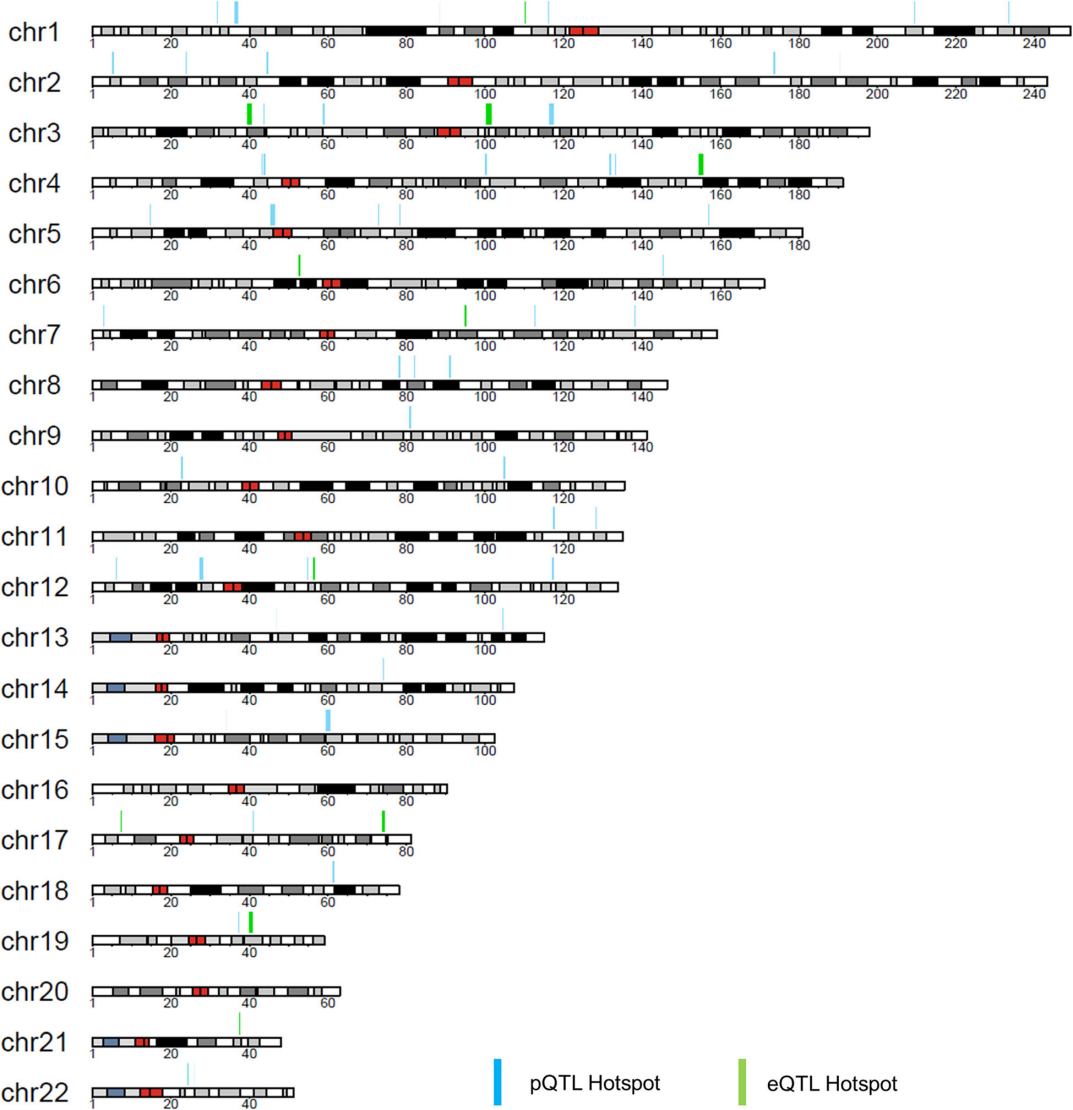
Genome hotspots for pQTL and eQTL identified in human livers. A pQTL hotspot was identified where genome distance between pQTLs is less than 1 Mb, and the Spearman’s rho values of the associated proteins were > 0.5 or < − 0.5. eQTL hotspots were identified with the same method

Protein expression correlation analysis revealed that 1358 out of the 1508 quantified proteins correlated with the expressions of other proteins (Spearman’s rho > 0.5 or < − 0.5). We identified 53 pQTL hotspots for these correlated proteins (Fig. 4, Additional file 8: Data file S3). The largest one is hotspot 22 (chr5: 45320127-46298172), while the smallest one is hotspot 2 (chr2: 190452249-190452250). Of note, the pQTLs in hotspot 53 (chr22: 25781394-25781897) were associated with expressions of 12 proteins, and all of them are involved in mitochondrial ATP synthesis. The median value of Spearman’s rho among proteins associated with pQTLs in hotspots was 0.826 (Additional file 9: Fig. S5), which is significantly higher than the median value of Spearman’s rho (0.146) between all proteins. Moreover, shared hotspots are significantly enriched (*p* value 2.2 × 10^−16^) in highly correlated protein pairs (Spearman’s rho > 0.5 or < − 0.5) relative to protein pairs with low correlations (Spearman’s rho < 0.5 and > − 0.5). These results suggest that pQTLs in the hotspots might be involved in some essential regulatory mechanisms of protein co-expression.

We also identified 12 eQTL hotspots for the correlated proteins (Spearman’s rho > 0.5 or < − 0.5) using published human liver eQTL data [8–11] (Additional file 8: Data file S3). Interestingly, one eQTL hotspot (chr22: 24237463-24240340) was also a pQTL hotspot. The e/pQTLs in this hotspot were associated with both mRNA and protein expressions of DDT and MIF.

### Networks regulating protein expression in human livers

**Fig. 5.**
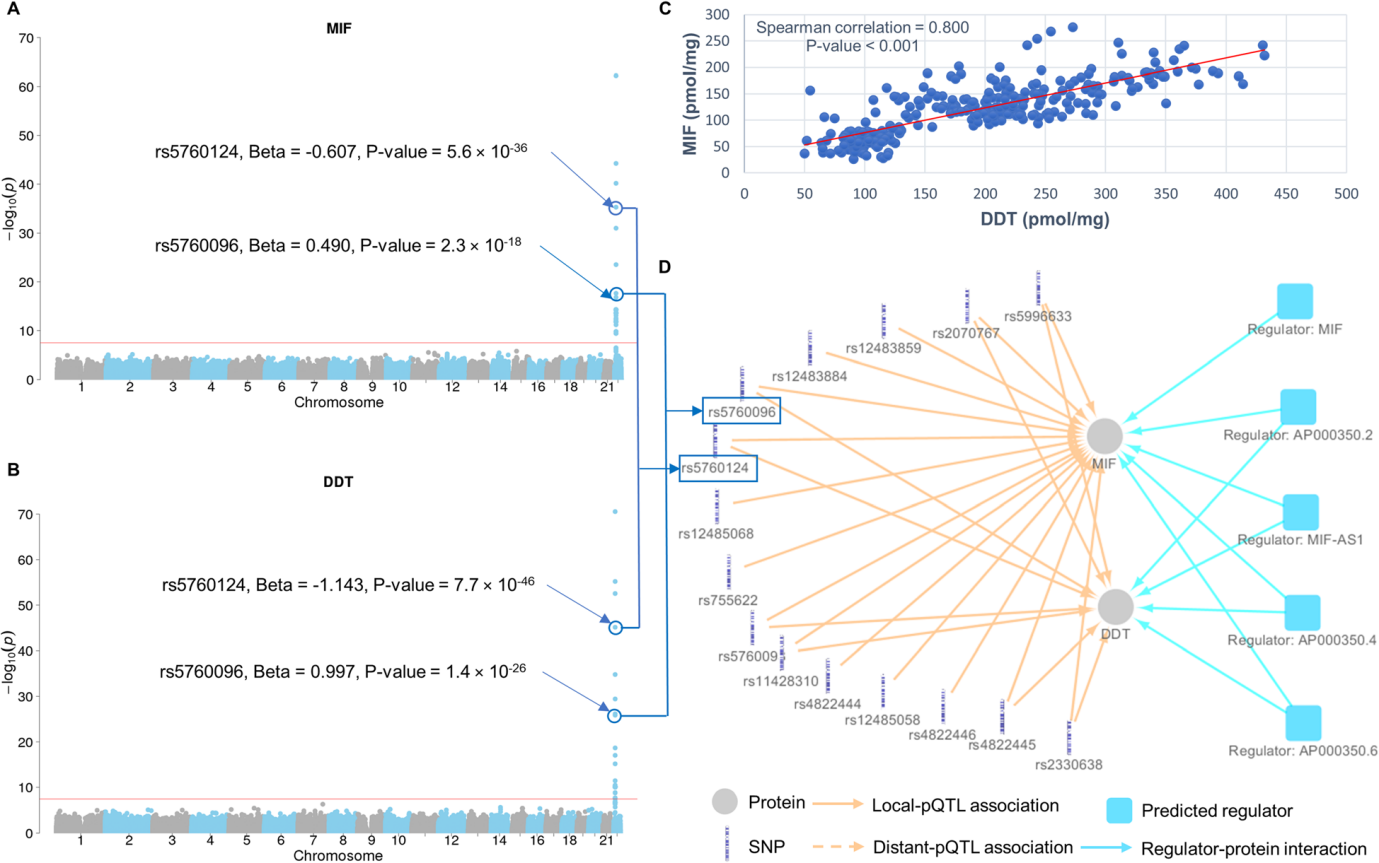
Protein regulatory networks for MIF and DDT. Manhattan plots of pQTL variants associated with protein levels of MIF (**a**) and DDT (**b**). Protein levels of MIF and DDT were highly correlated in human livers (**c**). Protein regulatory networks for DDT and MIF (**d**). The predicted regulators include protein-coding genes and non-coding RNA genes significantly enriched with pQTL variants (Bonferroni-adjusted *p* value < 0.05). Regulator nodes are formatted as “regulator: gene name”

To investigate the potential mechanisms underlying the associations between protein expression and pQTLs, especially the pQTLs in hotspots, we mapped the identified pQTL variants to transcriptional regulatory regions, such as promoters, CCCTC-binding factor (CTCF) binding sites, and enhancer regions. A total of 1133 pQTL variants were found to be located in these transcriptional regulatory regions (Additional file 4: Data file S2) and associated with the expression levels of 399 proteins. Among these, the metabolism of cobalamin associated B (MMAB) protein had the largest number of pQTL variants in the transcriptional regulatory regions, all of which were local pQTL (Additional file 4: Data file S2). Our results imply that transcriptional regulation of hepatic protein expression is a complex yet precisely regulated process, as exemplified by the pQTL variants of the macrophage migration inhibitory factor (MIF) (Fig. 5a) and d-dopachrome tautomerase (DDT) proteins (Fig. 5b), which appear to be co-regulated proteins associated with pQTLs in hotspots 51 and 52 (Additional file 8: Data file S3). DDT is a cytokine and a functional homolog of MIF [18]. Levels of MIF and DDT proteins were highly correlated (Spearman’s rho = 0.800, *p* value < 0.001, Fig. 5c), and they shared eight local pQTL variants including rs5760096 and rs5760124 (Fig. 5d). rs5760096 is located in a TF binding site in an enhancer region and positively associated with the expression of both DDT (beta = 0.997, *p* value = 1.4 × 10^−26^) and MIF (beta = 0.490, *p* value = 2.3 × 10^−18^) (Fig. 5a, b, Additional file 4: Data file S2). rs5760124 resides in a CTCF binding site in an open chromatin region and was negatively associated with the expression of both DDT (beta = − 1.143, *p* value = 7.7 × 10^−46^) and MIF (beta = − 0.607, *p* value = 5.6 × 10^−36^) (Fig. 5a, b; Additional file 4: Data file S2). The binding of CTCF to an open chromatin region can form a potent inhibitor of transcription [19]. Thus, an antagonistic transcriptional mechanism is likely involved in the regulation of MIF and DDT protein expression. This unique regulatory mechanism might be necessary for more precise expression control, given that the two proteins need to work in concert in various biological processes [20].

**Fig. 6.**
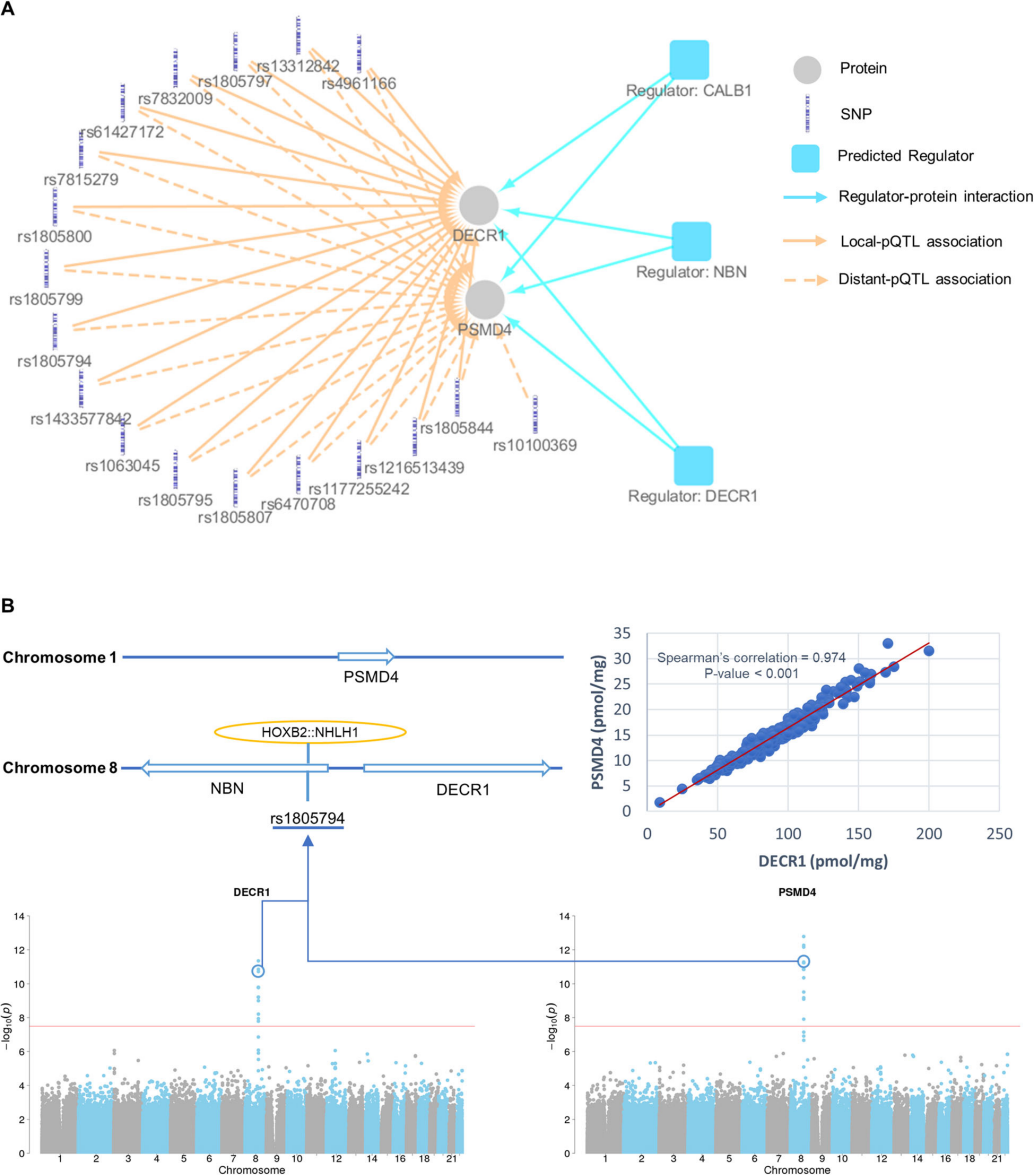
Protein regulatory networks for DECR1 and PSMD4. Protein regulatory networks for DECR1 and PSMD4 (**a**). The predicted regulators include protein-coding genes and non-coding RNA genes significantly enriched with pQTL variants (Bonferroni-adjusted *p* value < 0.05). Predicted regulator nodes are formatted as “regulator: gene name”. Co-regulation of DECR1 and PSMD4 in human livers (**b**). Chromosome positions of *NBN*, *DECR1*, and *PSMD4* (**b**, top-left). Genomic region enrichment analysis predicted NBN to be a regulator of DECR1 and PSMD4. Protein levels of DECR1 and PSMD4 were highly correlated in human livers (**b**, top-right). Manhattan plots of pQTL variants associated with DECR1 (**b**, bottom-left) and PSMD4 (**b**, bottom-right). The missense variant rs1805794 of NBN is in a binding site of the HOXB2::NHLH1 transcription factor complex (**b**, top-left). The rs1805794 was also one of the top pQTL variants significantly associated with the protein expressions of DECR1 (**b,** bottom-left) and PSMD4 (**b,** bottom-right)

Another interesting example of the protein expression regulation network can be found in the regulation of 2,4-dienoyl-CoA reductase 1 (DECR1) and proteasome 26S subunit, non-ATPase 4 (PSMD4) protein expression, which are co-regulated proteins associated with pQTLs in hotspots 32 and 33. DECR1 and PSMD4 shared 17 pQTL variants, all of which were local pQTLs for DECR1 but distant pQTLs for PSMD4 (Fig. 6a).

In addition to transcriptional regulatory factor analysis, we conducted a genomic region enrichment analysis to further investigate the potential regulators of protein expression. The analysis predicted 595 regulators within protein-coding genes and non-coding RNA genes for 394 proteins (Additional file 10: Data file S4). The UTRs, CDS, and exonic and intronic regions of the predicted regulators were significantly enriched with pQTL variants (Bonferroni-adjusted *p* value < 0.05). As an example, we found pQTL variants for MIF and DDT to be enriched in the exon regions of several non-coding RNA genes, such as *AP000350.2*, *MIF-AS1*, and *AP000350.6* (Fig. 5d). Besides non-coding RNA genes, some protein-coding genes were also enriched for pQTL variants. For example, pQTL variants of DECR1 and PSMD4 were significantly enriched (Bonferroni-adjusted *p* value < 0.05) in the UTRs, exons, and introns of the nibrin (NBN) gene (Fig. 6a). DECR1 and PSMD4 were almost perfectly correlated (Spearman’s rho = 0.974, *p* value < 0.001) (Fig. 6b, top-right) and shared 29 pQTL variants (Additional file 4: Data file S2). Of these pQTL variants, rs1805794 is a missense variant of *NBN* (Fig. 6b, top-left). Meanwhile, rs1805794 is within the binding site of the HOXB2:NHLH1 transcription factor complex and was one of the top pQTL variants negatively associated with protein levels of DECR1 (beta = − 0.535, *p* value = 1.6 × 10^−10^) and PSMD4 (beta = − 0.110, *p* value = 5.8 × 10^−12^) (Fig. 6b). These results imply potential relationships between NBN, DECR1, and PSMD4.

### Genome-wide pQTL analysis filled the gap between variants and associated traits

**Fig. 7.**
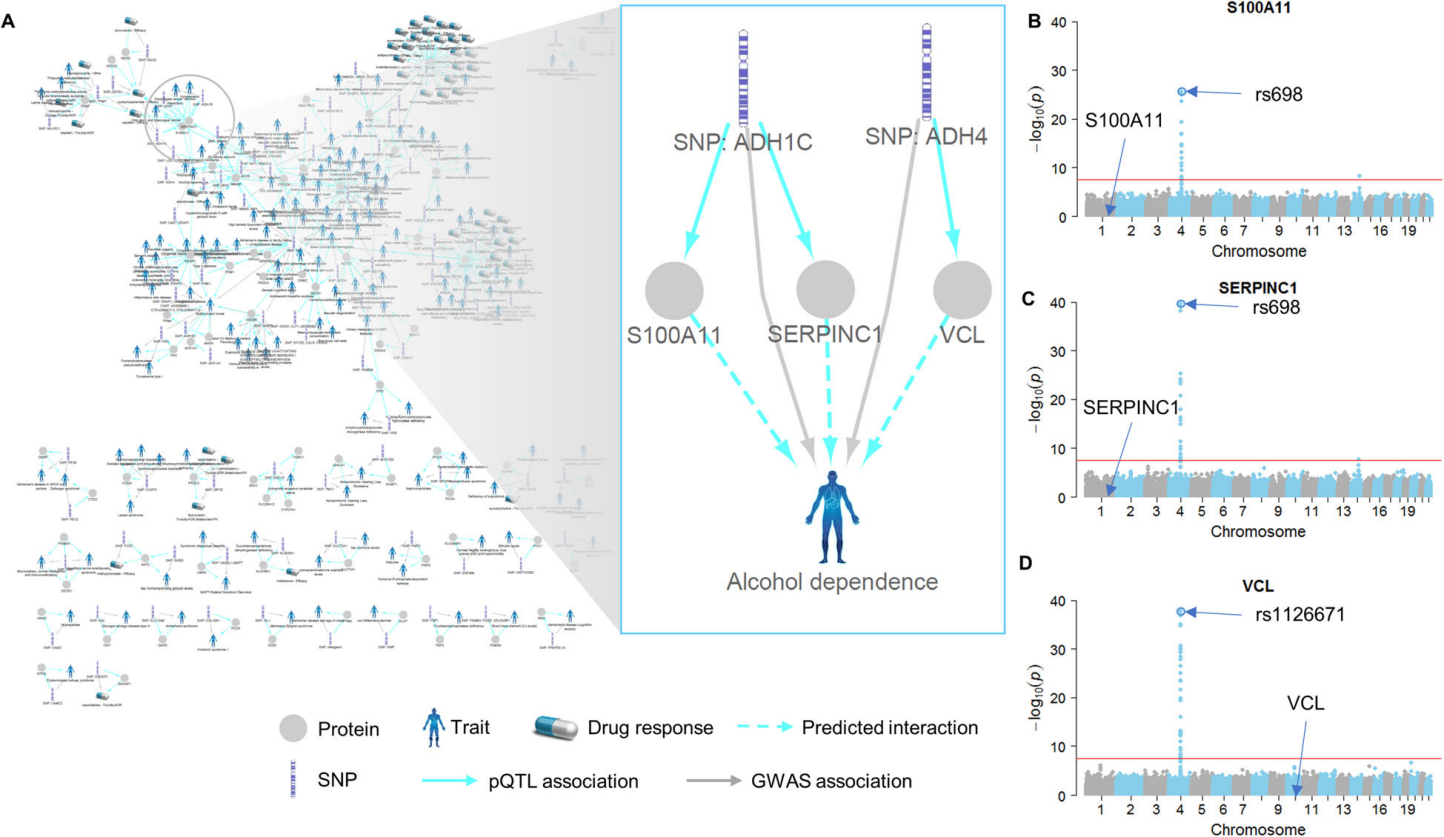
Genome-wide pQTL analysis filled the gap between genome variants and associated traits. Interaction networks of pQTL variants, proteins, and traits in human livers (**a**). The variant-protein-trait interactions were discovered by pQTL and variant-trait integration analysis. Variant-protein, variant-trait, and protein-trait interactions are marked as blue solid, gray solid, and blue dotted lines, respectively. Variants located in the same gene are combined and named as “SNP: gene name”. Manhattan plot of pQTL variants associated with S100A11 (**b**), SERPINC1 (**c**), and VCL (**d**)

To date, tens of thousands of associations between genetic variants and clinical traits, such as diseases and drug responses, have been uncovered by numerous association studies. However, understanding the molecular mechanisms underlying the observed associations remains a challenging task. Given that pQTLs are more predictive of gene functions than eQTLs, we expect that pQTL analysis can help reveal the mechanisms governing the interactions between genetic variants and clinical traits. Accordingly, we collected published association data from GWAS Catalog, ClinVar, PharmGKB databases, and mapped pQTLs to clinical traits via rs numbers. We found that 131 pQTL variants mapped to 201 traits (Additional file 11: Data file S5), suggesting 252 protein-trait interactions (Fig. 7a). The results shed light on the mechanisms underlying the associations between SNPs and clinical traits. For example, rs698 is a missense variant in alcohol dehydrogenase 1C (*ADH1C*), which is involved in alcohol metabolism [21]. This variant is associated with susceptibility to alcohol dependence [22]. In this study, rs698 was found to be a distant pQTL variant negatively associated with protein expression levels of S100 calcium-binding protein A11 (S100A11) and serpin family C member 1 (SERPINC1) (Fig. 7b, c; Additional file 4: Data file S2), and S100A11and SERPINC1 are potential co-regulated proteins associated with pQTLs in hotspots 17 and 18 (Additional file 8: Data file S3). Our results suggest that S100A11 and SERPINC1 may be involved in molecular mechanisms underlying the association between rs698 and alcohol dependence.

We further performed a colocalization analysis of primary pQTL and GWAS signals and found that the lead pQTL variant of AKR1A1 and HAAO were colocalized with the GWAS variants associated with blood protein levels and hypospadias, respectively (Additional file 7: Table S2).

## Discussion

As the largest internal organ in the human body, the liver is involved in numerous critical physiological functions, such as digestion and detoxification. Precise regulation of gene expression is essential for these functions. For the first time, we conducted a genome-wide pQTL study to reveal networks regulating hepatic protein expression in humans. Several studies have identified thousands of eQTL variants in human livers [8–11]. The comparison between our pQTL and previous eQTL findings revealed that only a small portion of QTL variants were associated with both protein and mRNA expressions, and all of which were local QTLs. Distant QTLs were associated with either mRNA or protein levels. Transcriptional regulation often relies on the binding of transcription factors to a genome position close to the regulated gene, which makes eQTLs more likely to be local regulatory elements. Therefore, relative to pQTL analysis, eQTL studies usually employ more stringent criteria to identify distant regulatory variants [8–11]. Thus, the discrepancy in distant variants between previous eQTL and our pQTL analysis could be in part due to the differences in data analysis approaches. However, the greater number of distant pQTLs identified in our pQTL study may reflect the fact that protein levels can be affected by additional post-transcriptional and post-translational regulatory mechanisms, in contract to mRNA levels. The observation suggests the importance of studying the gene expression at the protein level in order to comprehend the phenotypes of genetic variants.

In the present study, we discovered 53 genome hotspots containing pQTLs for 1358 correlated proteins. Interestingly, pQTLs for the correlated proteins were grouped to form a “hotspot” on the genome. Proteins associated with pQTLs in a hotspot often share the same function or belong to the same biological pathway (Additional file 8: Data file S3). For example, pQTLs in hotspot 53 were associated with the expressions of 12 proteins, including ATP synthase, transporters, and electron transfer flavoprotein dehydrogenase. All these proteins are involved in the mitochondrial ATP synthesis pathway, suggesting that the hotspot analysis could help uncover the co-regulating mechanisms of proteins in a specific biological process. However, we must take precautions when interpreting the results from the hotspot analysis. For example, a hotspot may result from a variant of a transcription factor that regulates the expression of multiple functionally unrelated genes.

This study discovered both transcriptional and post-transcriptional mechanisms involved in regulating protein expression in human livers (Additional file 4: Data file S2 and Additional file 10: Data file S4). The data can help understand the protein co-regulation mechanisms involving variants in pQTL hotspots. For example, MIF and DDT protein expressions are associated with pQTLs in hotspots 51 and 52. Our results suggest that their protein levels were not only transcriptionally regulated by the local pQTLs rs5760096 and rs5760124 but may also post-transcriptionally regulated by the non-coding RNAs *AP000350.2*, *MIF-AS1*, and *AP000350.6* (Fig. 5d). Both rs5760096 and rs5760124 are located in TF binding sites, but their effects were in opposite directions. *AP000350.2* is a kelch-like 5 (KLHL5) pseudogene and produces a non-coding RNA. *MIF-AS1* encodes a non-coding antisense RNA. *AP000350.6* encodes a long intergenic non-coding RNA (lincRNAs). The pQTL variants associated with DDT and MIF were significantly enriched (Bonferroni-adjusted *p* value < 0.05) in exon regions of these three non-coding RNA genes (Fig. 5d, Additional file 10: Data file S4). Thus, the DDT and MIF protein levels appear to be co-regulated by these transcriptional and post-transcriptional regulators, leading to a highly correlated expression pattern (Spearman’s rho = 0.783, *p* value < 0.001) in human livers. The regulation of DDT and MIF co-expression demonstrates that hepatic protein expression is precisely and efficiently regulated through a complex regulation network at the transcriptional and post-translational levels.

The complexity of protein expression regulation can be further exemplified by the co-regulation of the *DECR1* and *PSMD4* genes respectively located on chromosome 8 and 1. DECR1 and PSMD4 protein levels associated with pQTLs in hotspots 32 and 33 (Additional file 8: Data file S3). Their protein levels were almost perfectly correlated (Spearman’s rho = 0.974, *p* value < 0.001) (Fig. 6b, top-right) and shared 29 pQTL variants, of which 17 pQTL variants were in transcription regulatory regions, 11 pQTL variants were in coding genes, and 1 pQTL variant was in an intergenic region (Additional file 4: Data file S2 and Additional file 10: Data file S4). The enrichment analysis of pQTL variants suggests that the *NBN* gene is a potential regulator of both DECR1 and PSMD4 (Fig. 6a). Since NBN protein was undetectable due to its low expression level in the liver [23], we were unable to determine the association between NBN protein expression and the protein levels of DECR1 and PSMD4. However, the biological functions of the three proteins indicate a potential protein-protein interaction among NBN, DECR1, and PSMD4. NBN is a component of the MRE11-RAD50-NBN (MRN) complex, which plays a critical role in the early steps of cellular response to DNA damage and repair [24, 25]. DECR1 participates in the beta-oxidation [26]. PSMD4 is a component of the 26S proteasome, which is involved in the ATP-dependent degradation of ubiquitinated proteins and DNA damage response [27]. Both DECR1 and PSMD4 are involved in biological progresses initiated by NBN in response to DNA damage [28], indicating that DECR1 and PSMD4 could be the downstream functional proteins of NBN for DNA damage repair. Of the identified pQTL variants, rs1805794 is a missense variant of *NBN* (Fig. 6b, top-left), and the variant was associated with altered NBN functions and various diseases [29–31]. Interestingly, rs1805794 also resides in the transcription factor binding site of *DECR1* and *PSMD4*, suggesting that this SNP may directly impact the transcription of *DECR1* and *PSMD4*. However, functional experiments are needed to verify whether rs1805794 is involved in the co-regulation of these three genes.

This study not only uncovered protein regulatory networks but also linked proteins to specific clinical traits, such as diseases and drug responses, through integrated pQTL and GWAS analysis (Additional file 11: Data file S5). For instance, we were able to link S100A11 and SERPINC1, proteins associated with pQTLs in hotspots 17 and 18, to alcohol dependence through their distant pQTL variants located in *ADH1C* (Additional file 11: Data file S5, Fig. 7a). ADH1C protein is involved in alcohol metabolism and associated with alcohol dependence [22, 32]. S100A11 belongs to the S100 protein family and is a known inflammatory factor [33]. SERPINC1 is a member of the serpin superfamily and a plasma protease inhibitor; it inhibits thrombin and acts as an anti-inflammatory factor [34]. Inflammation is important for the development of alcohol dependence [35]. However, the origin and molecular mechanisms of inflammation in alcohol dependence remain unclear. The study implies that inflammation associated with the development of alcohol dependence might be partially mediated by S100A11 and SERPINC1 proteins.

This study also revealed a potential role for metabolites in the trans-regulation of protein production in human livers. The SNP rs1126671, a missense variant in *ADH4*, was found to be a distant pQTL variant for vinculin (VCL) (Fig. 7d). ADH4 is a member of the alcohol dehydrogenase family and participates in the retinoid metabolism [36]; retinoid can induce significant expression of VCL [37]. Therefore, rs1126671 may affect the VCL protein expression by altering the retinoid metabolism via its effect on ADH4 activity. Since the liver is the largest source of metabolic enzymes, this conclusion leads to the hypothesis that metabolites could act as intermediates for distant pQTLs in the regulation of protein expression in human livers.

## Conclusions

In sum, for the first time, protein expression regulation networks have been proposed in human livers via a global absolute quantitative proteomics-based pQTL analysis. The expression of hepatic proteins was found to be tightly regulated by both transcriptional and post-transcriptional regulatory elements in a complex yet precise manner. This study has suggested many post-translational regulatory elements, such as non-coding RNAs, and protein-protein interactions, which would be impossible using conventional eQTL approaches. We also discovered that pQTLs formed many hotspots on the genome, which may contribute to the co-expression of proteins. Furthermore, the study sheds light on our understanding of the mechanisms through which genetic variants contribute to clinical traits. Finally, the wealth of data generated by the study (Additional files 2: Data file S1, 4: Data file S2, 8: Data file S3, 10: Data file S4, 11: Data file S5) provides a valuable resource for the scientific community of investigators in the field of hepatology research. Future functional experiments would be critical to validate these findings.

## Materials and methods

### Human liver samples

We obtained normal human liver tissues from three providers: XenoTech LLC (Lenexa, KS, USA), the University of Minnesota Liver Tissue Cell Distribution System, and the Cooperative Human Tissue Network (CHTN). We randomly selected a subset of samples from the banked tissues for this investigation. The demographic information of the donors is limited, and we summarized the gender and ethnicity information in Supplementary Table S3 (Additional file 12). To avoid possible *p* value inflation caused by population structure, we performed a genotype principal component analysis to identify outliers (Additional file 12: Fig. S6) and included 287 samples in the final pQTL analysis.

### Liver S9 fraction preparation

We prepared human liver S9 fractions (HLS9) from about 200 mg of frozen liver tissues using a previously published method [38, 39]. Briefly, we cut liver tissues into small pieces (1 × 1 × 1 mm) and homogenized them using a microcentrifuge pestle in 1.5-mL microcentrifuge tubes with 0.5 mL ice-cold phosphate-buffered saline (PBS). We centrifuged the homogenates at 9000*g* for 20 min at 4 °C, and the top lipid-containing layer was removed. This centrifugation and top layer removal were repeated once, and the resulting supernatants (S9 fractions) were collected and stored at − 80 °C until analysis.

### Data-independent acquisition proteomics

We prepared HLS9 samples for proteomic analysis using the method detailed previously [39]. Briefly, following protein reduction and alkylation, we first digested protein samples using lysyl endopeptidase (protein to lysyl endopeptidase = 100:1) in an orbital incubator shaker at 220 rpm and 37 °C for 6 h. We then performed the second step digestion using tosyl phenylalanyl chloromethyl ketone-treated trypsin at a protein to trypsin ratio of 50:1 at 220 rpm and 37 °C overnight. We conducted the LC-MS/MS analysis on a Sciex TripleTOF 5600 plus mass spectrometer system coupled with an Eksigent 2D plus LC system. We used a trap-elute LC configuration for sample separation, which included a trapping column (ChromXP C18-CL, 120 Å, 5 μm, 10 × 0.3 mm, Eksigent Technologies) and an analytical column (ChromXP C18-CL, 120 Å, 150 × 0.3 mm, 5 μm, Eksigent Technologies). The mobile phase consisted of water with 0.1% formic acid (phase A) and acetonitrile containing 0.1% formic acid (phase B). The sample was first trapped and cleaned on the trapping column with the mobile phase A delivered at a flow rate of 10 μL/min for 3 min before being separated on the analytical column with a gradient at a flow rate of 5 μL/min. The gradient program was set as follows for the mobile phase B: 0–68 min, 3–30%; 68–73 min, 30–40%; 73–75 min, 40–80%; 75–78 min, 80; 78–79 min, 80–3%; and 79–90 min, 3%. We injected 6 μg of peptides into the mass spectrometer and also included an injection of 6 μL of water between each sample to minimize sample carryover. The mass spectrometer was operated in positive ion mode with the ion spray voltage floating at 5500 V and the source temperature set at 280 °C. The DIA scheme included a 250-ms TOF-MS scan from 400 to 1250 Da and MS/MS scans from 100 to 1500 Da [39]. The MS/MS scans of all precursors were performed in a cyclic manner using a 100-variable isolation window scheme. The accumulation time was 25 ms per isolation window, resulting in a total cycle time of 2.8 s. We used the Spectronaut™ Pulsar software (version 11.0, Biognosys AG, Schlieren, Switzerland) to obtain MS2 signals of fragment ions from DIA data with default settings (precursor *Q* value < 0.01, protein *Q* value < 0.01) with an in-house reference spectral library generated in our previous study [17]. To generate this library, we used the MaxQuant (version: 1.5.3, Max Planck Institute of Biochemistry, Martinsried, Germany) software to analyze the data-dependent acquisition (DDA) data of HLS9 samples. We selected trypsin as the digestion enzyme, set peptide-to-spectrum match (PSM) false discovery rate (FDR) < 0.01 and protein FDR < 0.01, and selected the “match between runs” during the MaxQuant analysis. We used a human reference proteome FASTA file containing 21,010 protein entries and 74,856 additional protein isoforms downloaded from Uniprot on February 1, 2018.

### Absolute protein quantification

Absolute protein expression levels in human livers were determined by the DIA-TPA method we recently published using MS2 intensity signals obtained from DIA [17]. We calculated the concentration of protein *i* [pmol] in 1 mg total input protein with the following equation:

$$ \mathrm{Protein}(i)=\frac{\mathrm{MS}2\ \mathrm{signal}(i)}{\mathrm{Total}\ \mathrm{MS}2\ \mathrm{signal}\times \mathrm{Molecular}\ \mathrm{mass}(i)}\times {10}^9 $$

The MS2 signal(*i*) is the sum of the MS2 peak areas of all detected peptides from protein *i*. Total MS2 signal is the sum of the MS2 peak areas of all peptides reported by Spectronaut™ Pulsar under default settings (Precursor *Q* value < 0.01). Molecular mass(*i*) is the molecular weight of protein *i*. Note that some peptides were shared by different proteins. We reasoned that the relative MS signals from unique peptides of different proteins would reflect the relative abundances of the individual proteins, and thus, the MS signals of shared peptides can be correctly distributed among the proteins based on the relative abundances of unique peptides. Accordingly, we calculated MS2 signal(*i*) for proteins with shared peptides using the following equation:

$$ \mathrm{MS}2\ \mathrm{signal}(i)=\sum \mathrm{MS}2\ \mathrm{signal}{(i)}_{\mathrm{unique}}+\sum \frac{\sum \mathrm{MS}2\ \mathrm{signal}{(i)}_{\mathrm{unique}}}{\sum \mathrm{MS}2\ \mathrm{signal}{(G)}_{\mathrm{unique}}}\mathrm{MS}2\ \mathrm{signal}{(G)}_{\mathrm{Shared}} $$

In this equation, ∑MS2 signal(*i*)_unique_ is the sum of the MS2 peak areas of unique peptides from protein *i*, and ∑MS2 signal(*G*)_unique_ is the sum of the MS2 peak areas of peptides unique to a group of proteins that have shared peptides with protein *i*. MS2 signal(*G*)_Shared_ is the MS2 peak areas of all peptides shared between protein *i* and other proteins in the group. Therefore, $\sum \frac{\sum \mathrm{MS}2\ \mathrm{signal}{(i)}_{\mathrm{unique}}}{\sum \mathrm{MS}2\ \mathrm{signal}{(G)}_{\mathrm{unique}}}\mathrm{MS}2\ \mathrm{signal}{(G)}_{\mathrm{Shared}}$ is the redistribution of the MS2 peak areas of the shared peptides to protein *i*.

### Subcellular location and protein expression correlation

We obtained protein subcellular locations from Gene Ontology (GO) annotation data downloaded from the GO database on January 24, 2019. We mapped proteins to subcellular locations using Uniprot IDs. We used R package *Hmisc* to determine Spearman’s rank correlations (Spearman’s rho) between different protein expressions. We plotted protein correlation using the Circos software.

### Genome-wide genotyping

We genotyped the 287 human liver samples using the Illumina Multi-Ethnic Global Array (Illumina, Miami, USA). This genotyping array contains 1,779,819 markers with comprehensive genomic coverage for several global populations. We used Genome Studio (Illumina, Miami, USA) for SNP calling, while subsequent quality control (QC) analysis was performed using PLINK (version 1.9). We removed SNPs with call rates < 0.99, with MAF < 0.01, or deviating from Hardy-Weinberg equilibrium (*p* < 0.0001) from the dataset.

### Genotype imputation

We used the Michigan Imputation Server (https://imputationserver.sph.umich.edu) to impute SNPs that passed QC. The Imputation Server is based on the Minimac3 algorithm and the 1000 Genomes Project cosmopolitan reference panel (Phase 3 v5) [40]. QC analysis was applied to the imputed genotypes using PLINK (version 1.9) to remove SNPs having an estimated posterior probability lower than 0.99 in any of the 287 samples, a call rate < 0.99, MAF < 0.01, or deviation from Hardy-Weinberg equilibrium with *p* < 0.0001. SNPs on sex chromosomes were excluded from the analysis.

### pQTL analysis

The genome-wide association study (GWAS) for pQTL analysis in human livers included a dataset of 1,671,387 SNPs merged from the genotyping array and imputation analysis. We performed linkage disequilibrium (LD) pruning utilizing PLINK (version 1.9) with window size 50, step size 5, and *R*^2^ threshold 0.8. The analysis identified 803,444 SNPs as independent locus markers. We quantified absolute protein expression levels of 1508 proteins in 287 human liver samples using the aforementioned DIA-TPA proteomic method. Among those, 1344 proteins were quantifiable in more than 90% of human liver samples and were included in the pQTL analysis. We determined the additive effects of each SNP on protein expression utilizing PLINK (version 1.9) with a linear regression model, which used ethnicity, gender, and the top three principal components identified from the genotype principal component analysis (Additional file 12: Fig. S6) as covariates. We obtained ethnicity and gender information from sample providers. To account for multiple comparisons, we used a *p* value threshold of 2.99 × 10^−8^ based on the number of the SNPs analyzed, which is equivalent to a FDR of ~ 1%. pQTL variants within 1 Mb of the gene being regulated were considered local pQTLs, while other pQTL variants were defined as distant pQTLs [41, 42]. The estimated false discovery rates of the local pQTLs and distant pQTLs were 0.005% and 1.3%, respectively. We generated Manhattan plots of the GWAS results using the R package *qqman*.

### pQTL hotspot analysis

Two neighboring pQTL variants were selected as a hotspot seed if (1) the two variants were associated with the expressions of different proteins, (2) the distance of the variants was less than 1 Mb, and (3) the absolute value of Spearman’s correlation (Spearman’s rho) among the proteins associated with the variants was over 0.5. Another neighboring pQTL variant was added to the hotspot if the variant was within 1 Mb from the hotspot seed, and the absolute value of Spearman’s correlation between the protein associated with the candidate variant and the protein associated with the nearest variant in the hotspot seed was over 0.5. We repeated this step until no neighboring pQTL variant met the above criteria. To investigate the function of every hotspot region, we selected GO annotations annotated to at least half of proteins in every single hotspot as the annotations of the hotspot. We performed the enrichment analysis of shared hotpots among highly correlated protein pairs (Spearman’s rho > 0.5 or < − 0.5) and protein pairs with low correlations (Spearman’s rho < 0.5 and > − 0.5) using the Fisher exact test.

### eQTL data and eQTL hotspot analysis

We retrieved eQTL data directly from previous studies [8–11] without performing additional eQTL analysis. We remove eQTLfrom the datasets if (1) a SNP was not genotyped in our study and (2) eQTL is for a transcript that was not detected by our proteomics assay. We identified eQTL hotspots using the same method and criteria for pQTL hotspots as described above.

### Transcriptional regulatory region mapping of pQTL

We downloaded data describing the transcriptional regulatory regions and binding motif features from the Ensembl database (release 95). The data included 217,681 CTCF binding sites, 140,648 enhancers, 119,409 open chromatin regions, 149,918 promoters, 110,191 promoter flanking regions, and 385,381,574 TF binding sites. We mapped the identified pQTL variants to these regions based on their chromosome positions in the Genome Reference Consortium Human Build 37 (GRCh37).

### Genomic region enrichment of pQTL

We mapped pQTL variants to UTRs, CDS, and the exons and introns of genes based on GRCh37 chromosome positions using the comprehensive gene annotation (v29lift37) downloaded from the GENCODE database. We used Fisher’s exact test to determine the enrichment of pQTL variants in UTRs, CDS, exons or introns of coding genes, and the exons of non-coding RNA genes. We adjusted the *p* values of the enrichment analysis using Bonferroni correction based on the number of tests. A significant enrichment was reported when the Bonferroni-adjusted *p* value was less than 0.05.

### Analysis of variant-trait associations

We downloaded published GWAS data from the GWAS Catalog database (v1.0.2), which includes 104,767 variant-trait associations. In addition, a total of 971,313 variant-trait associations were retrieved from the ClinVar database. We downloaded another 3938 variant-trait associations between variants and drug responses from PharmGKB. We mapped the identified pQTL variants to the traits via reference SNP cluster IDs (i.e., rs number).

### Colocalization analysis

We performed a colocalization analysis using the method similar to that implemented by Wu et al. [43]. For the colocalization of eQTL and pQTL signals, we were unable to determine lead eQTL variants that had the strongest evidence of association with mRNA expression because eQTLs were obtained from multiple studies. Therefore, we calculated pairwise LD *r*^*2*^ between every eQTL variants and lead pQTL variants that had the strongest evidence of association with protein expression. For variant pairs with LD *r*^*2*^ > 0.8, we tested the changes of the pQTL association for the lead pQTL variant when conditioned on the eQTL variant. We applied two criteria to define the colocalization of eQTL and pQTL: (1) lead variant pairwise *r*^2^ > 0.8 and (2) the *p* value of the lead pQTL variant to be no longer significant after conditional analysis. We further used the same method to perform the colocalization analysis of GWAS and pQTL signals.

## Supplementary information

**Additional file 1:** **Fig. S1.** Heatmap of quantified proteins in 287 human liver samples.

**Additional file 2:** **Data file S1.** Protein concentrations in the 287 human liver samples.

**Additional file 3:** **Fig. S2.** Features of the transcripts of the genes with quantifiable protein expression in the human liver eQTL study. Violin plots of log10-transformed protein concentrations in major subcellular locations (**A**). The analysis was performed using subcellular location data obtained from the Gene Ontology (GO) database. Violin plot of Spearman’s correlations (Spearman’s Rho) of transcript levels in human liver (**B**). The medium value of Spearman’s Rho was about 0.354. Transcriptome data were obtained from GTEx. We only analyzed the transcripts of the genes with quantifiable protein expression in the present human liver eQTL study.

**Additional file 4:** **Data file S2.** pQTL variants discovered in the human livers.

**Additional file 5:** **Fig. S3.** Statistics of distant-pQTLs and local-pQTLs. Distant-pQTLs (**A left**) and local-pQTLs (**B left**) found to be associated with various number of proteins. Violin plots of number of distant-pQTLs (**A right**) and local-pQTLs (**B right**) per protein.

**Additional file 6:** **Fig. S4.** Comparison of eQTLs and pQTLs in human livers. eQTL data were obtained from published eQTL studies. Venn plots of eQTL and pQTL associated genes (**A**), eQTL and pQTL associations (**B**), and eQTL and pQTL variants (**C**). Distribution of local- and distant-QTL variants across genomic regions (**D**: eQTLs and **E**: pQTLs).

**Additional file 7:** **Table S1.** Colocalization of pQTL and eQTL signals. Additional file 7: Table S2. Colocalization of pQTL and GWAS signals. Additional file 7: Table S3. Gender and ethnicity of human liver samples.

**Additional file 8:** **Data file S3.** Genome hotspots for p/eQTL identified in the human livers.

**Additional file 9:** **Fig. S5.** Violin plot of Spearman’s correlations (Spearman’s Rho) of protein expression levels for hotspot proteins. Correlations of proteins associated with pQTLs in a same hotspot were shown in this plot.

**Additional file 10:** **Data file S4.** Regulators of hepatic proteins predicted by genomic enrichment analysis of pQTL variants. Predicted regulators include protein coding genes and non-coding RNA genes.

**Additional file 11:** **Data file S5.** Interactions among pQTL variants, proteins, and traits. The pQTL variant-protein interactions were discovered by pQTL analysis. The variant-trait interactions were obtained from the GWAS Catalog, ClinVar and PharmGKB databases. The protein-trait interactions were discovered by the integrated pQTL and trait-variants analysis.

**Additional file 12:** **Fig. S6.** The first three principal components (PCs) analysis of the genotypes of the 287 human liver samples. The L274 was the outlier in the PC1 and PC2 analysis, L161 and L464 were the outliers in the PC1 and PC3 analysis, and L81, L464 and L274 were the outliers in the PC2 and PC3 analysis. However, there were no outlier samples in all three PC analyses.

## Data Availability

All data needed to evaluate this work are present in the paper and/or the Supplementary Materials. All LC-MS/MS data have been deposited to the ProteomeXchange Consortium with the dataset identifier PXD019169 [44]. Additional data related to this paper may be requested from the authors.

## References

[1] Lee SI, Dudley AM, Drubin D, Silver PA, Krogan NJ, Pe’er D, Koller D (2009). Learning a prior on regulatory potential from eQTL data. PLoS Genet.

[2] Strunz T, Grassmann F, Gayan J, Nahkuri S, Souza-Costa D, Maugeais C, Fauser S, Nogoceke E, Weber BHF (2018). A mega-analysis of expression quantitative trait loci (eQTL) provides insight into the regulatory architecture of gene expression variation in liver. Sci Rep.

[3] Battle A, Khan Z, Wang SH, Mitrano A, Ford MJ, Pritchard JK, Gilad Y (2015). Genomic variation. Impact of regulatory variation from RNA to protein. Science.

[4] Vogel C, Abreu Rde S, Ko D, Le SY, Shapiro BA, Burns SC, Sandhu D, Boutz DR, Marcotte EM, Penalva LO (2010). Sequence signatures and mRNA concentration can explain two-thirds of protein abundance variation in a human cell line. Mol Syst Biol.

[5] Vogel C, Marcotte EM (2012). Insights into the regulation of protein abundance from proteomic and transcriptomic analyses. Nat Rev Genet.

[6] Chick JM, Munger SC, Simecek P, Huttlin EL, Choi K, Gatti DM, Raghupathy N, Svenson KL, Churchill GA, Gygi SP (2016). Defining the consequences of genetic variation on a proteome-wide scale. Nature.

[7] Williams EG, Wu Y, Jha P, Dubuis S, Blattmann P, Argmann CA, Houten SM, Amariuta T, Wolski W, Zamboni N (2016). Systems proteomics of liver mitochondria function. Science.

[8] Schadt EE, Molony C, Chudin E, Hao K, Yang X, Lum PY, Kasarskis A, Zhang B, Wang S, Suver C (2008). Mapping the genetic architecture of gene expression in human liver. PLoS Biol.

[9] Schroder A, Klein K, Winter S, Schwab M, Bonin M, Zell A, Zanger UM (2013). Genomics of ADME gene expression: mapping expression quantitative trait loci relevant for absorption, distribution, metabolism and excretion of drugs in human liver. Pharmacogenomics J.

[10] GTExConsortium (2017). Genetic effects on gene expression across human tissues. Nature.

[11] Innocenti F, Cooper GM, Stanaway IB, Gamazon ER, Smith JD, Mirkov S, Ramirez J, Liu W, Lin YS, Moloney C (2011). Identification, replication, and functional fine-mapping of expression quantitative trait loci in primary human liver tissue. PLoS Genet.

[12] Xie F, Liu T, Qian WJ, Petyuk VA, Smith RD (2011). Liquid chromatography-mass spectrometry-based quantitative proteomics. J Biol Chem.

[13] Schwanhausser B, Busse D, Li N, Dittmar G, Schuchhardt J, Wolf J, Chen W, Selbach M (2011). Global quantification of mammalian gene expression control. Nature.

[14] Anand S, Samuel M, Ang CS, Keerthikumar S, Mathivanan S (2017). Label-based and label-free strategies for protein quantitation. Methods Mol Biol.

[15] Zhu X, Chen Y, Subramanian R (2014). Comparison of information-dependent acquisition, SWATH, and MS^All^ techniques in metabolite identification study employing ultrahigh-performance liquid chromatography-quadrupole time-of-flight mass spectrometry. Anal Chem.

[16] Schubert OT, Ludwig C, Kogadeeva M, Zimmermann M, Rosenberger G, Gengenbacher M, Gillet LC, Collins BC, Rost HL, Kaufmann SH (2015). Absolute proteome composition and dynamics during dormancy and resuscitation of mycobacterium tuberculosis. Cell Host Microbe.

[17] He B, Shi J, Wang X, Jiang H, Zhu HJ (2019). Label-free absolute protein quantification with data-independent acquisition. J Proteome.

[18] Merk M, Zierow S, Leng L, Das R, Du X, Schulte W, Fan J, Lue H, Chen Y, Xiong H (2011). The D-dopachrome tautomerase (DDT) gene product is a cytokine and functional homolog of macrophage migration inhibitory factor (MIF). Proc Natl Acad Sci U S A.

[19] Song L, Zhang Z, Grasfeder LL, Boyle AP, Giresi PG, Lee BK, Sheffield NC, Graf S, Huss M, Keefe D (2011). Open chromatin defined by DNaseI and FAIRE identifies regulatory elements that shape cell-type identity. Genome Res.

[20] Gunther S, Fagone P, Jalce G, Atanasov AG, Guignabert C, Nicoletti F (2019). Role of MIF and D-DT in immune-inflammatory, autoimmune, and chronic respiratory diseases: from pathogenic factors to therapeutic targets. Drug Discov Today.

[21] Martinez C, Galvan S, Garcia-Martin E, Ramos MI, Gutierrez-Martin Y, Agundez JA (2010). Variability in ethanol biodisposition in whites is modulated by polymorphisms in the ADH1B and ADH1C genes. Hepatology.

[22] Biernacka JM, Geske JR, Schneekloth TD, Frye MA, Cunningham JM, Choi DS, Tapp CL, Lewis BR, Drews MS, LP T (2013). Replication of genome wide association studies of alcohol dependence: support for association with variation in ADH1C. PLoS One.

[23] Fagerberg L, Hallstrom BM, Oksvold P, Kampf C, Djureinovic D, Odeberg J, Habuka M, Tahmasebpoor S, Danielsson A, Edlund K (2014). Analysis of the human tissue-specific expression by genome-wide integration of transcriptomics and antibody-based proteomics. Mol Cell Proteomics.

[24] D’Amours D, Jackson SP (2002). The Mre11 complex: at the crossroads of DNA repair and checkpoint signalling. Nat Rev Mol Cell Biol.

[25] Kobayashi J, Antoccia A, Tauchi H, Matsuura S, Komatsu K (2004). NBS1 and its functional role in the DNA damage response. DNA Repair (Amst).

[26] Yu W, Chu X, Chen G, Li D (2005). Studies of human mitochondrial 2,4-dienoyl-CoA reductase. Arch Biochem Biophys.

[27] Jacquemont C, Taniguchi T (2007). Proteasome function is required for DNA damage response and fanconi anemia pathway activation. Cancer Res.

[28] Chatterjee N, Walker GC (2017). Mechanisms of DNA damage, repair, and mutagenesis. Environ Mol Mutagen.

[29] Silva J, Teixeira AL, Lobo F, Mauricio J, Medeiros R (2012). DNA repair system and prostate cancer progression: the role of NBS1 polymorphism (rs1805794). DNA Cell Biol.

[30] Jin G, Wang M, Chen W, Shi W, Yin J, Gang W (2015). Single nucleotide polymorphisms of nucleotide excision repair and homologous recombination repair pathways and their role in the risk of osteosarcoma. Pak J Med Sci.

[31] Zhang H, Liu Y, Zhou K, Zhou C, Zhou R, Cheng C, Wei Q, Lu D, Zhou L (2016). Genetic variations in the homologous recombination repair pathway genes modify risk of glioma. J Neuro-Oncol.

[32] Treutlein J, Cichon S, Ridinger M, Wodarz N, Soyka M, Zill P, Maier W, Moessner R, Gaebel W, Dahmen N (2009). Genome-wide association study of alcohol dependence. Arch Gen Psychiatry.

[33] Andres Cerezo L, Sumova B, Prajzlerova K, Veigl D, Damgaard D, Nielsen CH, Pavelka K, Vencovsky J, Senolt L (2017). Calgizzarin (S100A11): a novel inflammatory mediator associated with disease activity of rheumatoid arthritis. Arthritis Res Ther.

[34] Levy JH, Sniecinski RM, Welsby IJ, Levi M (2016). Antithrombin: anti-inflammatory properties and clinical applications. Thromb Haemost.

[35] Leclercq S, de Timary P, Delzenne NM, Starkel P (2017). The link between inflammation, bugs, the intestine and the brain in alcohol dependence. Transl Psychiatry.

[36] Molotkov A, Deltour L, Foglio MH, Cuenca AE, Duester G (2002). Distinct retinoid metabolic functions for alcohol dehydrogenase genes Adh1 and Adh4 in protection against vitamin A toxicity or deficiency revealed in double null mutant mice. J Biol Chem.

[37] Helige C, Hofmann-Wellenhof R, Fink-Puches R, Smolle J (2004). Mofarotene-induced inhibition of melanoma cell motility by increasing vinculin-containing focal contacts. Melanoma Res.

[38] Wang X, Liang Y, Liu L, Shi J, Zhu HJ (2016). Targeted absolute quantitative proteomics with SILAC internal standards and unlabeled full-length protein calibrators (TAQSI). Rapid Commun Mass Spectrom.

[39] Shi J, Wang X, Lyu L, Jiang H, Zhu HJ (2018). Comparison of protein expression between human livers and the hepatic cell lines HepG2, Hep3B, and Huh7 using SWATH and MRM-HR proteomics: focusing on drug-metabolizing enzymes. Drug Metab Pharmacokinet.

[40] Das S, Forer L, Schonherr S, Sidore C, Locke AE, Kwong A, Vrieze SI, Chew EY, Levy S, McGue M (2016). Next-generation genotype imputation service and methods. Nat Genet.

[41] Yao C, Chen G, Song C, Keefe J, Mendelson M, Huan T, Sun BB, Laser A, Maranville JC, Wu H (2018). Genome-wide mapping of plasma protein QTLs identifies putatively causal genes and pathways for cardiovascular disease. Nat Commun.

[42] Sun W, Kechris K, Jacobson S, Drummond MB, Hawkins GA, Yang J, Chen TH, Quibrera PM, Anderson W, Barr RG (2016). Common genetic polymorphisms influence blood biomarker measurements in COPD. PLoS Genet.

[43] Wu Y, Broadaway KA, Raulerson CK, Scott LJ, Pan C, Ko A, He A, Tilford C, Fuchsberger C, Locke AE (2019). Colocalization of GWAS and eQTL signals at loci with multiple signals identifies additional candidate genes for body fat distribution. Hum Mol Genet.

[44] He B, Shi J, Wang X, Jiang H, Zhu HJ: Genome-wide pQTL analysis of protein expression regulatory networks in the human liver. ProteomeXchange (www.proteomexchange.org), Identifier: PXD019169. Accessed 14 May 2020. 10.1186/s12915-020-00830-3 PMC7418398 32778093

