# Supplementary figures and images for: Genome-wide pQTL analysis of protein expression regulatory networks in the human liver
Source: BMC Biol. 2020 Aug 10;18:97. doi: 10.1186/s12915-020-00830-3 (PMC7418398; doi:10.1186/s12915-020-00830-3)

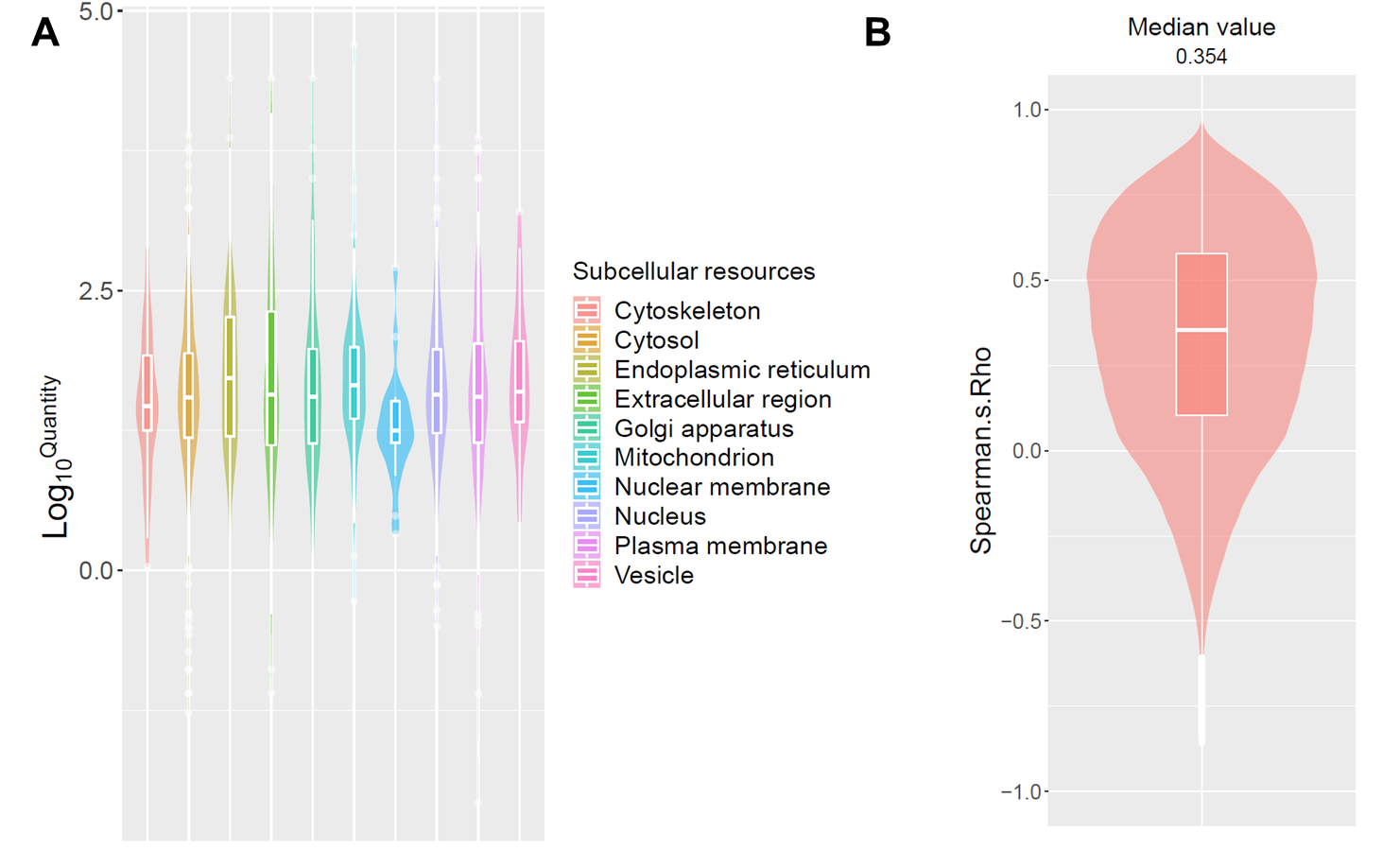

Supplement: Supplementary file 3 — Additional file 3: Fig. S2. Features of the transcripts of the genes with quantifiable protein expression in the human liver eQTL study. Violin plots of log10-transformed protein concentrations in major subcellular locations (A). The analysis was performed using subcellular location data obtained from the Gene Ontology (GO) database. Violin plot of Spearman’s correlations (Spearman’s Rho) of transcript levels in human liver (B). The medium value of Spearman’s Rho was about 0.354. Transcriptome data were obtained from GTEx. We only analyzed the transcripts of the genes with quantifiable protein expression in the present human liver eQTL study. [file 12915_2020_830_MOESM3_ESM.tif]

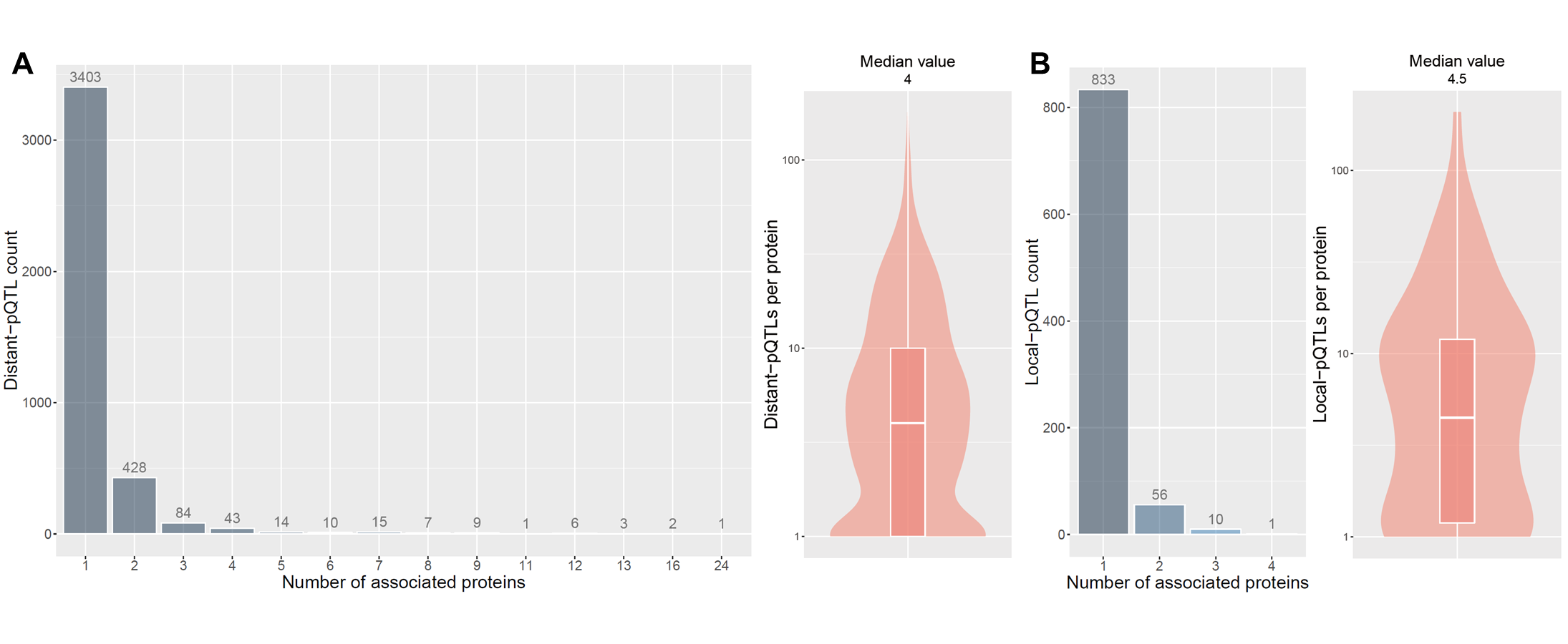

Supplement: Supplementary file 5 — Additional file 5: Fig. S3. Statistics of distant-pQTLs and local-pQTLs. Distant-pQTLs (A left) and local-pQTLs (B left) found to be associated with various number of proteins. Violin plots of number of distant-pQTLs (A right) and local-pQTLs (B right) per protein. [file 12915_2020_830_MOESM5_ESM.tif]

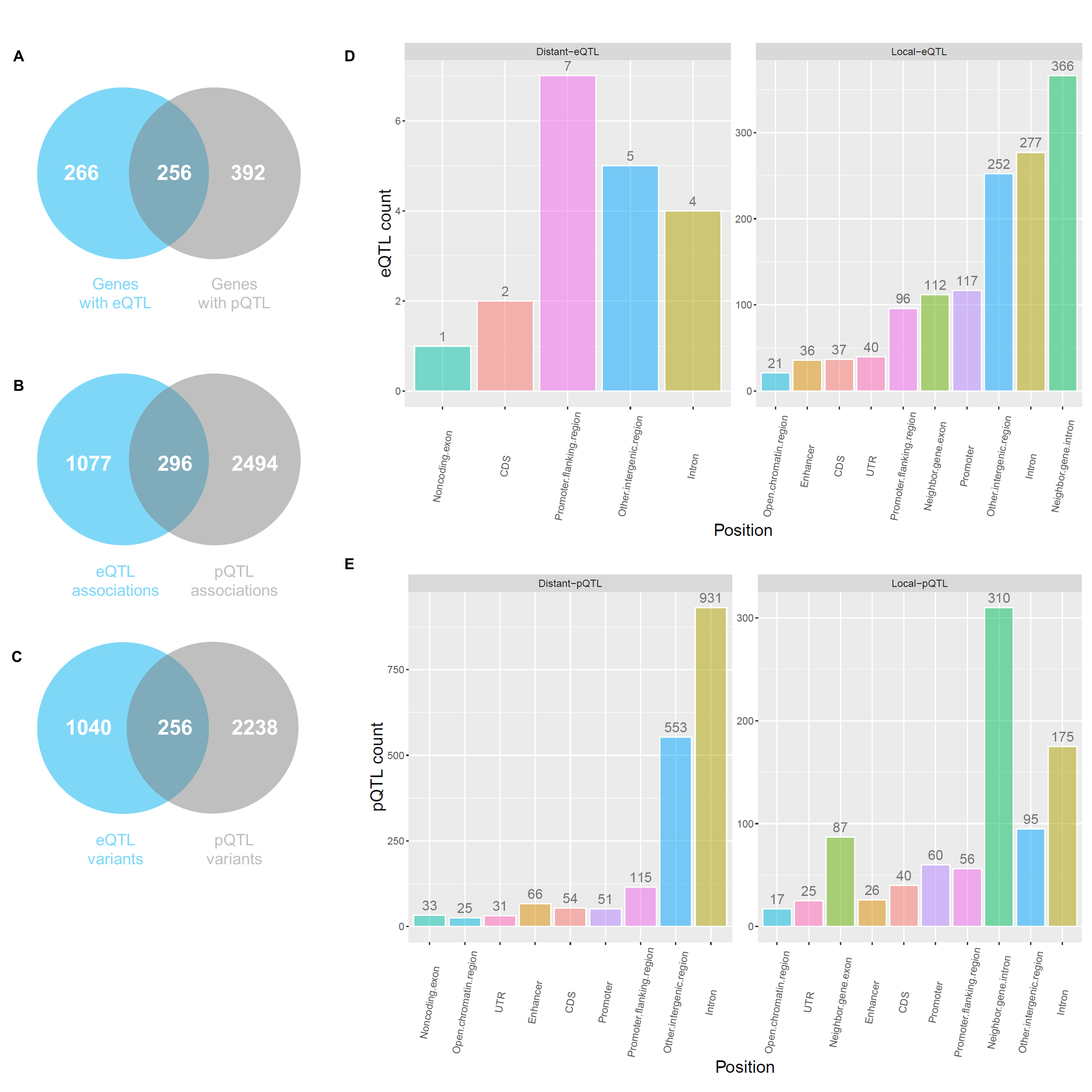

Supplement: Supplementary file 6 — Additional file 6: Fig. S4. Comparison of eQTLs and pQTLs in human livers. eQTL data were obtained from published eQTL studies. Venn plots of eQTL and pQTL associated genes (A), eQTL and pQTL associations (B), and eQTL and pQTL variants (C). Distribution of local- and distant-QTL variants across genomic regions (D: eQTLs and E: pQTLs). [file 12915_2020_830_MOESM6_ESM.tif]

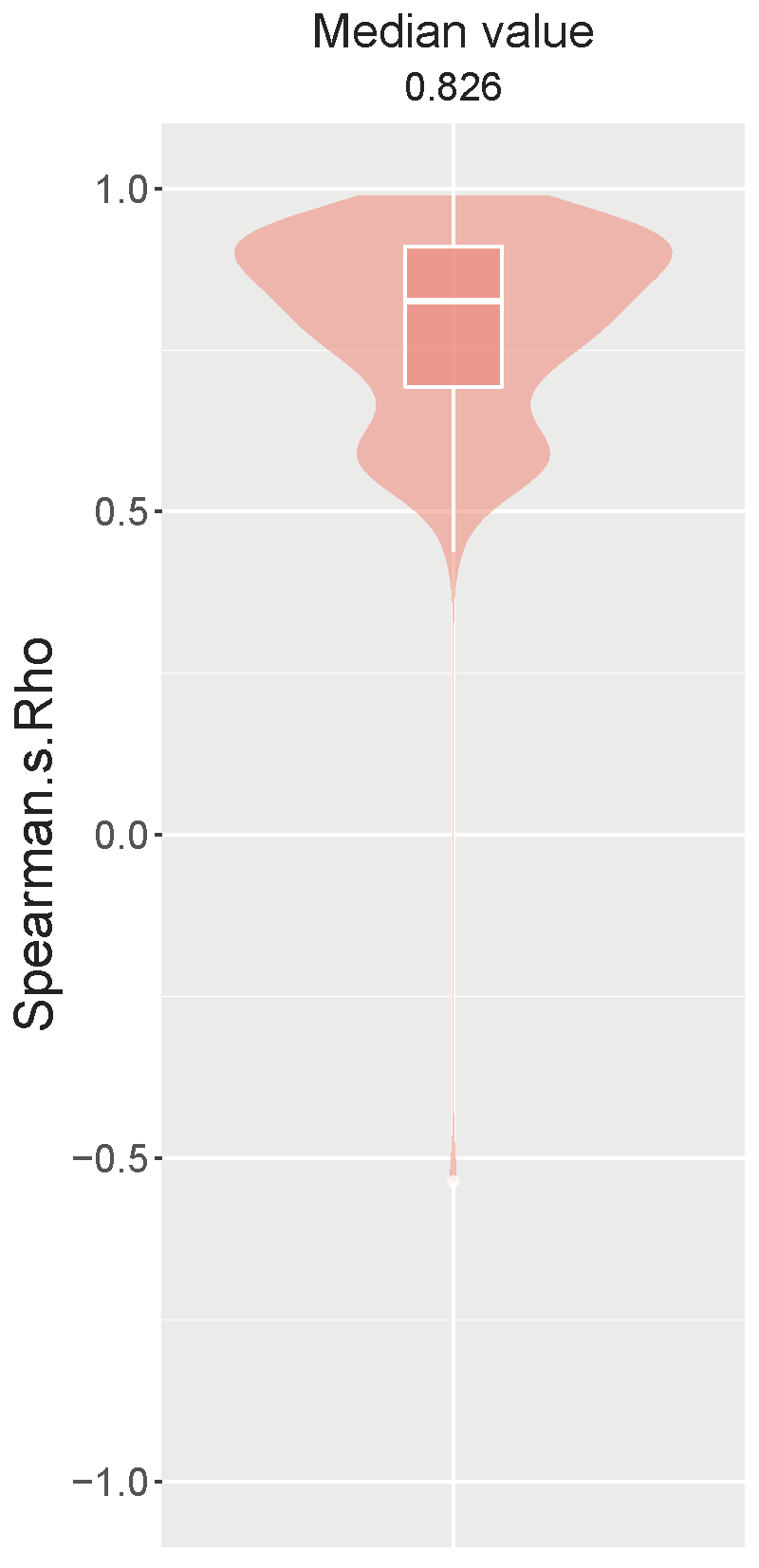

Supplement: Supplementary file 9 — Additional file 9: Fig. S5. Violin plot of Spearman’s correlations (Spearman’s Rho) of protein expression levels for hotspot proteins. Correlations of proteins associated with pQTLs in a same hotspot were shown in this plot. [file 12915_2020_830_MOESM9_ESM.tiff]

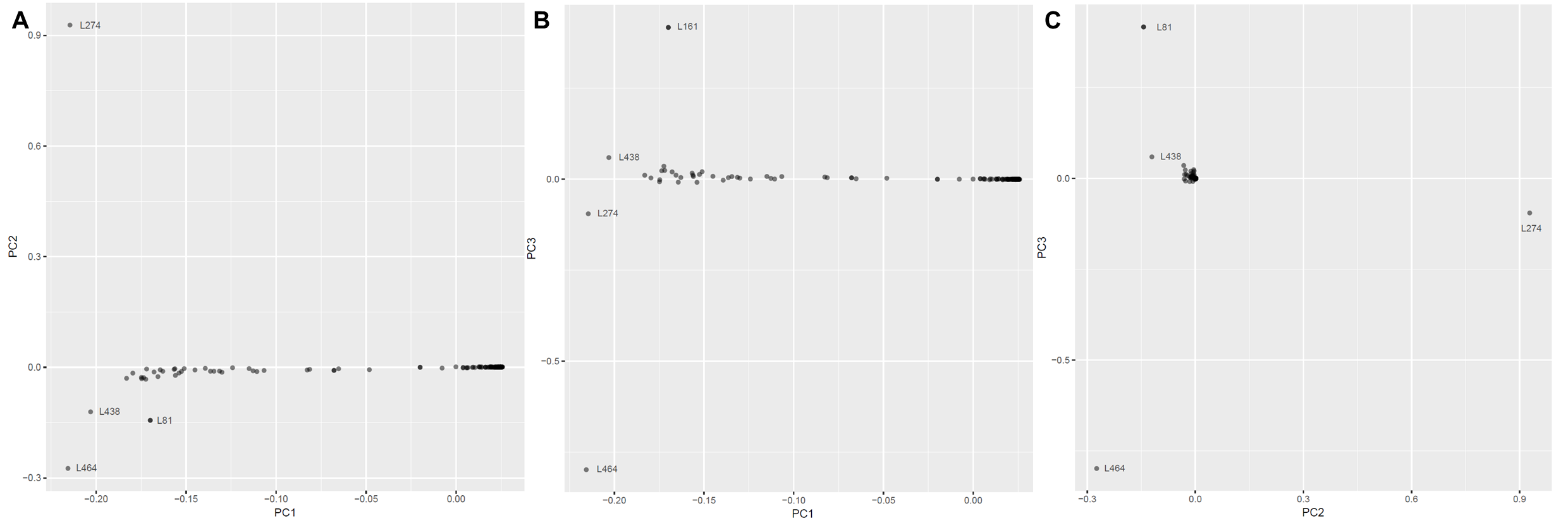

Supplement: Supplementary file 12 — Additional file 12: Fig. S6. The first three principal components (PCs) analysis of the genotypes of the 287 human liver samples. The L274 was the outlier in the PC1 and PC2 analysis, L161 and L464 were the outliers in the PC1 and PC3 analysis, and L81, L464 and L274 were the outliers in the PC2 and PC3 analysis. However, there were no outlier samples in all three PC analyses. [file 12915_2020_830_MOESM12_ESM.tif]
