# Supplementary material for: Genome-wide pQTL analysis of protein expression regulatory networks in the human liver
Source: BMC Biol. 2020 Aug 10;18:97. doi: 10.1186/s12915-020-00830-3 (PMC7418398; doi:10.1186/s12915-020-00830-3)
Supplement: Supplementary file 7 — Additional file 7: Table S1. Colocalization of pQTL and eQTL signals. Additional file 7: Table S2. Colocalization of pQTL and GWAS signals. Additional file 7: Table S3. Gender and ethnicity of human liver samples. [file 12915_2020_830_MOESM7_ESM.docx]

**Table S1. Colocalization of pQTL and eQTL signals.**

| **pQTL signal** | | **eQTL signal** | |
| --- | --- | --- | --- |
| **Lead pQTL variant** | **pQTL Protein** | **eQTL variant** | **eQTL Gene** |
| rs7652179 | GMPPB | rs7621797 | GMPPB |
| rs17842268  rs1204504  rs10562543 | QPRT | rs12447415 | QPRT |
|  | DECR2 | rs12443594 | DECR2 |
|  | ETHE1 | rs12980646 | ETHE1 |
| rs10562543  rs10562543  rs10562543  rs10562543 | ETHE1 | rs61607741 | ETHE1 |
|  | ETHE1 | rs59827264 | ETHE1 |
|  | ETHE1 | rs12981023 | ETHE1 |
|  | ETHE1 | rs59000923 | ETHE1 |
| rs893729 | HAAO | rs3816183 | HAAO |
| rs1884139 | DDAH2 | rs11161606 | DDAH1 |
| rs2608630 | GSTA2 | rs2749017 | GSTA2;GSTA1 |
| rs2608630 | GSTA2 | rs2144698 | GSTA2;GSTA1 |
| rs2608630 | GSTA2 | rs2608629 | GSTA2;GSTA7P;GSTA1 |
| rs2608630 | GSTA2 | rs2608632 | GSTA2;GSTA1 |
| rs2608630 | GSTA2 | rs2749016 | GSTA2;GSTA1 |
| rs2303463 | CNDP2 | rs12971120 | CNDP2 |

**Table S2. Colocalization of pQTL and GWAS signals.**

| **pQTL signal** | | **GWAS signal** | |
| --- | --- | --- | --- |
| **Lead pQTL variant** | **pQTL Protein** | **GWAS variant** | **Trait** |
| rs2229540 | AKR1A1 | rs72688441 | Blood protein levels |
| rs893729 | HAAO | rs3816183 | Hypospadias |

**Table S3. Gender and ethnicity of human liver samples.**

| **Characteristics** | | **Number of Samples** | **Percentage (%)** |
| --- | --- | --- | --- |
| **Gender** | Male | 114 | 39.72 |
|  | Female | 156 | 54.36 |
|  | Not available | 17 | 5.92 |
| **Ethnicity** | Caucasian | 172 | 59.93 |
|  | Black | 29 | 10.10 |
|  | Other | 3 | 1.05 |
|  | Not available | 83 | 28.92 |
