# Supplementary material for: Genome-wide pQTL analysis of protein expression regulatory networks in the human liver
Source: BMC Biol. 2020 Aug 10;18:97. doi: 10.1186/s12915-020-00830-3 (PMC7418398; doi:10.1186/s12915-020-00830-3)

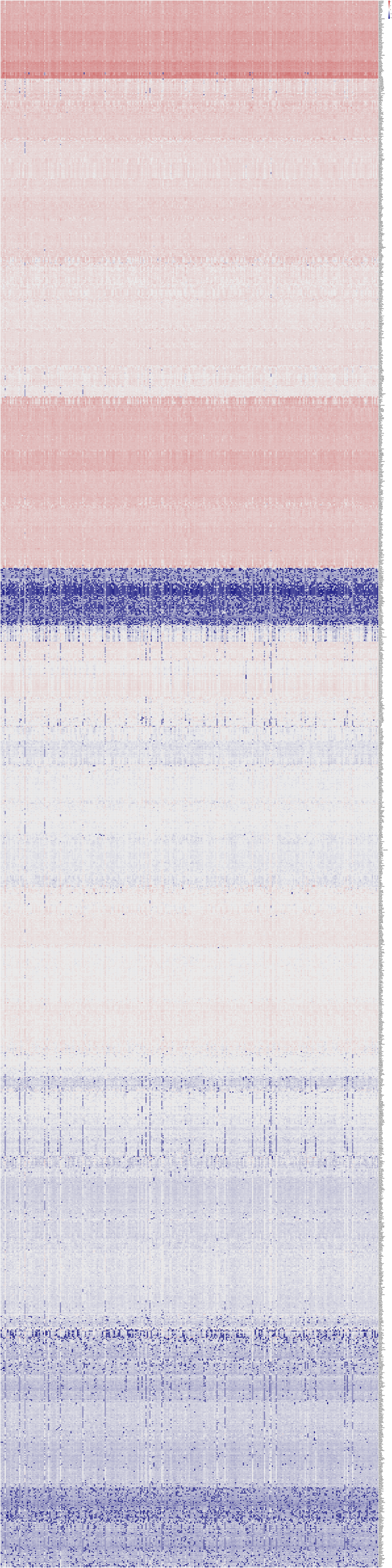

Supplement: Supplementary file 1 — Additional file 1: Fig. S1. Heatmap of quantified proteins in 287 human liver samples. [file 12915_2020_830_MOESM1_ESM.pdf]
